# Regulation of cortical activity and arousal by the matrix cells of the ventromedial thalamic nucleus

**DOI:** 10.1038/s41467-018-04497-x

**Published:** 2018-05-29

**Authors:** Sakiko Honjoh, Shuntaro Sasai, Shannon S Schiereck, Hirotaka Nagai, Giulio Tononi, Chiara Cirelli

**Affiliations:** 1 Department of Psychiatry, University of Wisconsin, Madison, WI USA; 2 Present Address: University of Tsukuba, International Institute for Integrative Sleep Medicine, Tsukuba, Ibaraki Japan

## Abstract

The “non-specific” ventromedial thalamic nucleus (VM) has long been considered a candidate for mediating cortical arousal due to its diffuse, superficial projections, but direct evidence was lacking. Here, we show in mice that the activity of VM calbindin1-positive matrix cells is high in wake and REM sleep and low in NREM sleep, and increases before cortical activity at the sleep-to-wake transition. Optogenetic stimulation of VM cells rapidly awoke all mice from NREM sleep and consistently caused EEG activation during slow wave anesthesia, while arousal did not occur from REM sleep. Conversely, chemogenetic inhibition of VM decreased wake duration. Optogenetic activation of the “specific” ventral posteromedial nucleus (VPM) did not cause arousal from either NREM or REM sleep. Thus, matrix cells in VM produce arousal and broad cortical activation during NREM sleep and slow wave anesthesia in a way that accounts for the effects classically attributed to “non-specific” thalamic nuclei.

## Abstract (web summary)

The ventromedial thalamus (VM) is thought to control cortical arousal through its diffuse projections to cortex. Here the authors record and manipulate the activity of calbindin1-positive matrix cells in VM and show that they bidirectionally regulate the sleep-wake transition.

## Introduction

The thalamus has long been involved in the regulation of cortical activity, arousal, attention, and sleep^1–5^. Early experiments in athalamic cats, for instance, found that extensive bilateral ablation of the thalamus with minimal cortical damage led to a complete dissociation between cortical activity and behavior, at least initially, with synchronous slow waves dominating the EEG during behavioral wakefulness, and to a permanent loss of spindle activity^6^. Ascribing a specific function to various thalamic nuclei, however, has been difficult, with a few notable exceptions, such as the definite role of the reticular thalamic nucleus in the generation of sleep spindles^7–9^. One reason for this difficulty is that classical lesion studies were either incomplete or too broad, involving passing fibers^10–12^. Moreover, our understanding of the organization of thalamic nuclei and cell types has undergone extensive revisions. The classical distinction was between specific thalamic nuclei projecting to a distinct cortical area and non-specific nuclei projecting diffusely to cortex and receiving diffuse afferents from the reticular formation of the brainstem. “Non-specific” midline and intralaminar nuclei are now subdivided, based on their patterns of connectivity, into at least four different groups^5^.

An important distinction is between two classes of thalamic neurons that project to the cerebral cortex, core, and matrix cells. Parvalbumin-positive core cells are enriched in sensory and motor relay nuclei and project mainly to layer 4 of specific cortical areas. Calbindin-positive matrix cells abound in some intralaminar and medial thalamic nuclei and project diffusely to cortex, primarily to superficial layers^13,14^. While core and matrix cells are often interspersed within many thalamic nuclei, these two cell classes exhibit cortical projection patterns typical of classical specific and non-specific systems, respectively. In particular, matrix cells within the medial thalamus may constitute a veritable thalamic activating system that facilitates effective interactions among many cortical areas and thereby sustains arousal, consciousness, and awareness of the environment^4,5,15,16^.

Until recently, it had not been possible to target matrix cells specifically. To do so, we employed a combination of anatomical, electrophysiological, optogenetic, and pharmacological experiments to test the specific role of matrix cells within the ventromedial nucleus (VM) of the thalamus. VM neurons qualify as multiareal matrix cells in the rat^17–19^: they project to almost the entire neocortex^20^, and each of them sends axon fibers to widespread cortical areas^21^. Unlike other non-specific nuclei with strong multilaminar patterns of projections, VM sends axons mainly to the upper part of layer 1 (L1)^21,22^, where they form relatively strong synapses preferentially into late-spiking (LS) L1 interneurons, as well as on distal apical dendrites of L2/3 and L5 pyramidal neurons^23^. LS interneurons correspond to neurogliaform cells, are strongly electrically coupled to each other, and inhibit both layer 2/3 pyramidal cells and non-LS cells fibers^23,24^. Because of their connectivity, VM cells are potentially in a good position to affect cortical activity strongly and diffusely. Consistent with this hypothesis, a 2-deoxy-glucose study found a widespread decline in cortical and thalamic metabolism after unilateral electrolytic lesions of VM, although aspecific effects due to damage of fibers of passage could not be excluded^25^. In this study, by recording and specifically stimulating VM neurons, we provide direct evidence for their role in cortical activation and arousal.

## Results

### Validation of Calb1-Cre mice

In the rat VM, matrix cells can be neurochemically identified because they express Calbindin 1 (Calb1 ^17^). Thus, we first examined the distribution of Calb1-positive cells of the mouse VM and their projection pattern. Endogenous Calb1 was expressed densely in VM, both in B6 mice and in Calb1-Cre mice (Supplementary Fig. 2A). Specifically, in the Calb1-Cre line Cre-dependent EYFP was expressed in most Calb1-positive cells (94.4 ± 1.7%, mean ± std; *n* = 3 mice) and no cells showed EYFP expression without Calb1, demonstrating high specificity and efficiency of the Cre recombinase activity. We then systematically quantified the cortical projections of EYFP-labeled VM neurons. These projections were widespread, targeted mainly layer 1 (L1) and especially the outer half of L1 (Supplementary Fig. 2B,C), and were densest in frontal regions, gradually decreasing along the A/P axis (Supplementary Fig. 2C). In parietal cortex (−1.8 A/P), secondary somatosensory cortex (S2) showed the strongest innervation (Supplementary Fig. 2C). Overall, these results in mice are highly consistent with previous evidence in other species showing that VM is comprised of Calb1-positive matrix cells that targets L1 of most cortical areas^17,21,22^.

### Activity pattern of VM neurons across the sleep/wake cycle

**Fig. 1:**
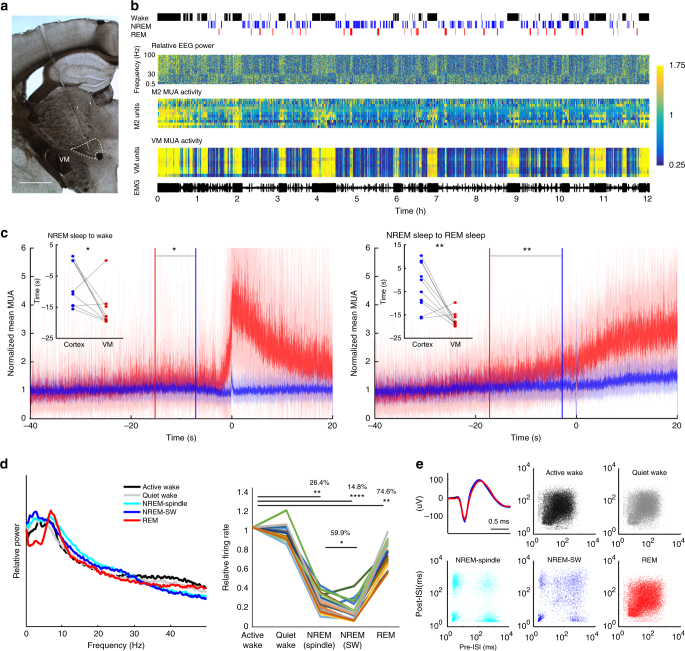
VM activity is state dependent. **a** Electrode placement for VM recording. Scale bar, 1 mm. **b** From top; representative hypnogram showing manually scored vigilance states during the light period. Relative EEG power in parietal cortex, normalized to the 12 h mean for each frequency (*Y* axis, 0.5–100 Hz, 1 Hz bin). MUA in M2 and VM (4-s epochs, normalized to the 12 h mean). Color bar represents relative changes for all three panels. M2 channels are arranged from superficial to deep layers. EMG electromyogram. **c** Mean MUA changes across state transitions (10 mice; M1 + M2 in blue, VM in red, solid line and shaded area; mean ± std). Each channel was averaged across the 12 h light period and values were normalized to the mean of baseline activity of each channel calculated between −40 and −20 s from time 0. Time 0 identifies the automatically detected awakening from NREM sleep based on EMG activity (left) or cortical activation at the onset of REM sleep (right, defined as the end of the last off period). The time interval between when MUA starts exceeding mean + 5 std of baseline and time 0 is shown for each mouse (dots) and averaged across all mice (blue and red vertical lines). Across mice MUA increased earlier in VM than in cortex (mean ± std in s, left; NREM-to-wake, M1 + M2 = −7.14 ± 7.29, VM = −15.91 ± 6.32, *p < 0.05, right, NREM-to-REM, M1 + M2 = −2.85 ± 9.84, VM = −17.23 ± 3.072, **p < 0.005, paired *t*-test). **d** Left, EEG power spectrum across vigilance states, for epochs used for spike sorting. Middle, mean VM firing rates normalized to active wake (each line is one neuron; 20 single units, 6 mice). *p < 0.01, **p < 5 × 10^−5^, ***p < 5 × 10^−6^, paired *t*-test). **e** Top left, representative waveforms of a VM single unit (average waveforms of 50 randomly selected spikes from each vigilance state). The other panels show 2D log-scale inter-spike interval scatterplots for the same VM neuron across vigilance states (*x* axis and *y* axis, interval in ms to previous and post spike, respectively)

To characterize Calb1-positive VM neurons electrophysiologically, we performed chronic recordings of cortical and thalamic activity in freely moving mice implanted with silicon probes in motor cortex (M1 or M2) and VM (Fig. 1a; *n* = 10 mice). During wake and REM sleep, cortical neurons are depolarized and fire asynchronously, resulting in low amplitude EEG and cortical LFP, while in NREM sleep they are bistable, alternating between depolarized (up) states when they fire (ON periods), and hyperpolarized (down) states when they are silent (OFF periods). Because ON and OFF periods are more or less synchronous across neurons, EEG and cortical LFP are dominated by slow wave activity (SWA) (0.5–4 Hz, Fig. 1b, Supplementary Fig. 3). VM neurons showed consistent and striking changes in firing patterns that were highly correlated with EEG changes, with sustained high firing during wake and REM sleep and low firing during NREM sleep (Fig. 1b, Supplementary Fig. 3). As expected, multi-unit activity (MUA) in cortex was also on average higher in wake and REM sleep than in NREM sleep, but sleep/wake changes in VM were more clear-cut and homogeneous across cells (Fig. 1b). We then focused on NREM sleep to wake transitions to establish the exact time when VM cells increase firing. We analyzed all awakenings from NREM sleep during the 12 h of the light period and found that in most mice (8/10) VM neurons increased firing several seconds before signs of cortical activation appeared and before the mouse started moving (Fig. 1c left, Supplementary Fig. 4). The same was true for the transitions from NREM sleep to REM sleep, when in most mice (8/10) firing in VM preceded cortical activation (Fig. 1c right, Supplementary Fig. 5).

To further assess VM neuronal activity we performed spike sorting and compared VM firing across active and quiet wake, NREM sleep with spindles, NREM sleep with slow waves, and REM sleep. These vigilance states differed, as expected, in their overall EEG power spectra (Fig. 1d, left). VM activity peaked during both active and quiet wake, dropped drastically in NREM sleep, especially when slow waves occurred, and reached 75% of the level seen in active wake during REM sleep (Fig. 1d right, Supplementary Fig. 3). These changes were highly consistent across VM neurons, suggesting that they represent a homogeneous population. Consistent with results in other thalamic neurons^15^, we also found that VM cells tend to fire tonically in wake and REM sleep and in burst mode in NREM sleep (Fig. 1e). Moreover, cross-correlogram analysis among VM neurons suggested highly synchronized firing in all behavioral states (Supplementary Fig. 6A,B). To measure the extent of this synchronization, we analyzed the peak distribution in cross-correlograms and identified synchronized pairs of neurons in either VM or cortex based on whether the corresponding cross-correlogram showed a peak above the mean + 5 std firing rate of the baseline period (−200 to −100 ms prior to the referential spike). Using this criterion, only 8–15% of all cortical neuron pairs showed synchronized activity, as opposed to more than 70% of VM neuron pairs (Supplementary Fig. 6C). Together with the widespread nature of the VM projections, these correlative results suggest that synchronous high activity of VM neurons can play a role in the activation of large portions of the cortex and across many layers.

### Optogenetic activation of VM induces arousal from NREM sleep

**Fig. 2:**
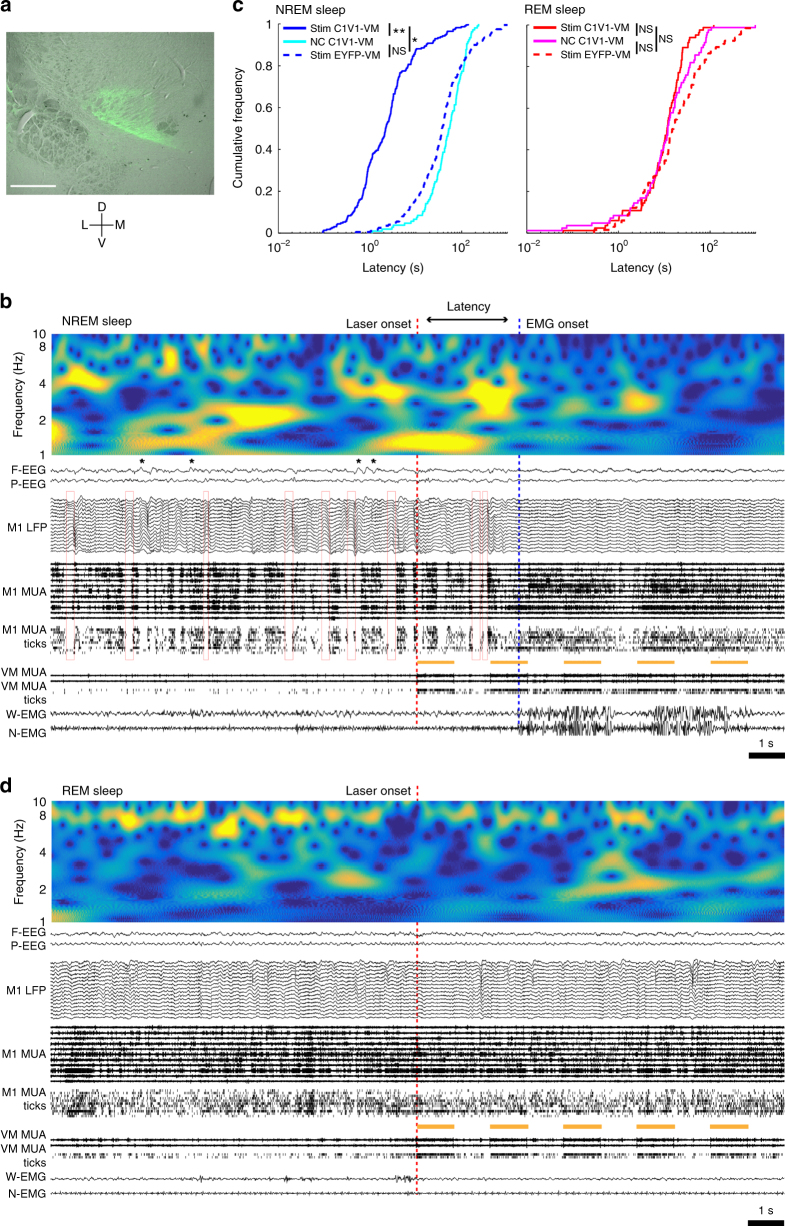
Fast C1V1-mediated optogenetic stimulation of VM neurons awakens mice from NREM sleep but not from REM sleep. **a** Coronal section of the mouse brain (Bregma −1.46 A/P) showing a representative example of virus expression in VM (DIO-C1V1-AAV5, green). Scale bar = 0.5 mm. **b** Example of awakening after laser stimulation of VM in NREM sleep, showing the 10 s before and after laser onset. Top; EEG power in the SWA (0.5–4.5 Hz) and theta (6–10 Hz) range decreases a few seconds after laser onset. Red open squares identify OFF periods. Yellow bars indicate laser pulses (five 1-s-laser pulses, spaced 1 s apart), which strongly drive VM unit firing (VM MUA and ticks, thresholded spikes). The blue dotted line indicates the time of manually scored awakening. **c** Cumulative distribution function (CDF) plots of automatically detected latency to awakening after VM stimulation in NREM and REM sleep in C1V1-VM mice. NS; *p* > 0.05, **p < 0.01, yellow light stimulation vs. negative control in C1V1-VM, paired *t*-test (*n* = 4 mice); *p < 0.05, yellow light stimulation in C1V1-VM vs. control mice, ks-test, using mean of each mouse (*n* = 4 mice/group). **d** Example of VM stimulation in REM sleep

To causally test the hypothesis that VM neurons may be important for vigilance state regulation, we optogenetically stimulated them during sleep and investigated cortical responses and behavioral consequences. The red-shifted channelrhodopsin variant C1V1 was injected bilaterally in Calb1-Cre mice to target Calb1-positive matrix cells in VM (Fig. 2a; *n* = 4 mice). Yellow light stimulation was delivered after at least 15 s of stable NREM sleep, characterized by slow waves in the EEG and LFP recordings and OFF periods in unit activity (Fig. 2b). The light pulses rapidly and reliably abolished all the hallmarks of NREM sleep and caused behavioral arousal in C1V1-VM mice, but not in controls. Specifically, C1V1-VM mice woke up within 10 s in more than 80% of cases, much faster than when either they received negative control stimulation or laser pulses were delivered to control (EYFP-VM) mice (Fig. 2b, c; Supplementary Fig. 7A,B). In C1V1-VM mice there was some variability in the latency to arousal after laser pulses, even if they were all delivered during polygraphically defined NREM sleep. Some of this variance could be explained by neuronal fatigue due to repeated stimulation, as the order in which laser pulses were delivered was positively correlated with latency (*R* = 0.22549, *p* < 0.001, Spearman correlation). Moreover, the latency was negatively correlated with SWA power in the last 10 s before stimulation (*R* = −0.19558, *p* < 0.003, Spearman correlation) and positively correlated with theta power during the same time window (*R* = 0.15846, *p* < 0.017, Spearman correlation), suggesting that even within NREM sleep the exact brain state at the time of stimulation also mattered. Specifically, mice woke up less promptly from a state relatively closer to REM sleep (low SWA, high theta). These results were obtained using the automatic detection of awakenings based on the whisker EMG signal, and were further confirmed when arousal was identified by manual visual scoring of the EEG traces (Supplementary Fig. 8A). We also tested laser pulses of two different frequencies, 25 Hz and 0.5 Hz, and both types of stimulation reliably elicited cortical activation and behavioral arousal (Supplementary Fig. 8B).

To further compare spontaneous and post-laser awakenings, spectral analysis was performed using cortical LFPs in M1 and M2, which are heavily targeted by VM. First, we identified spontaneous awakenings from NREM sleep during baseline and compared the 10 s before and after each awakening. In both M1 and M2, SWA (0.5–4 Hz) and theta power (6–10 Hz) decreased across all cortical layers at the transition from NREM sleep to wake, while high gamma power (80–100 Hz) increased, especially in the most superficial and the deepest layers (Supplementary Fig. 9A). Next, we performed the same analysis using 10 s before and after light onset delivered in NREM sleep, and found that when compared to the negative control condition (no stimulation), laser stimulation led to changes in LFP power similar to those seen in spontaneous NREM sleep to wake transitions, with decreases in both SWA and theta power (Supplementary Fig. 9A). Neither the same light stimulation in control mice (EYFP-VM), nor negative control stimulation in C1V1-VM mice affected LFP power (Supplementary Fig. 9A).

We then examined if activation of VM also causes arousal from REM sleep, which is characterized by an activated EEG with high theta activity and muscle atonia with phasic twitches, rapid eye movements and, in rodents, whisker movements. REM sleep is also called paradoxical sleep because cortical activity is wake-like, but the subjects are asleep and disconnected from environment, i.e., unable to respond to mild sensory stimuli. The same light stimulation that caused arousal from NREM sleep was ineffective when applied in REM sleep, with no obvious changes in EEG, LFP, MUA, and EMG signals (Fig. 2d) and no decrease in the latency to awakening (Fig. 2c). In our recordings a reliable marker of REM sleep was high theta activity especially in parietal cortex (Fig. 1d, Supplementary Fig. 3, Supplementary Fig. 5). At the spontaneous transition from REM sleep to wake, theta power decreased markedly in M1 and M2 across all layers, gamma power increased in the deep layers, and SWA increased in all layers of M2 and in the deep layers of M1 (Supplementary Fig. 9B). Optogenetic stimulation of VM slightly reduced theta power in the M1 middle layer and increased SWA power in M2, but deep layers in M1 and all layers in M2 showed no significant change in theta power (Supplementary Fig. 9B). As with NREM sleep, laser stimulation in EYFP-VM mice and control stimulation in C1V1-VM mice in REM sleep did not affect EEG power (Supplementary Fig. 9B). Thus, optogenetic stimulation of VM during REM sleep causes a decrease in theta activity restricted to some cortical layers, but did not result in behavioral arousal.

**Fig. 3:**
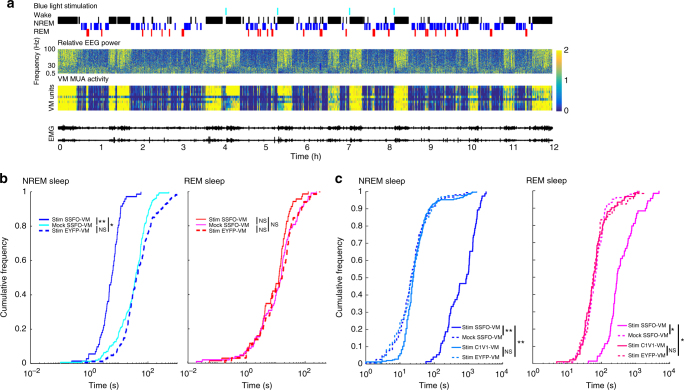
Prolonged SSFO-mediated activation of VM neurons awakens mice from NREM sleep and promotes sustained wakefulness. **a** Top; time of blue light stimulation and hypnogram showing manually scored vigilance states (4-s epochs; 12 h light period). All four blue light stimulations are followed by consolidated wake bouts. Relative EEG power in parietal cortex and VM MUA (4-s epochs) are normalized to the 12 h mean. **b** Cumulative distribution function (CDF) plots of automatically detected latency to awakening after VM stimulation in NREM and REM sleep in SSFO-VM mice and in control mice (GFP-VM). NS; *p* > 0.05, **p < 0.005, blue light stimulation vs. yellow mock stimulation in SSFO-VM, paired *t*-test (*n* = 4 mice); *p < 0.05, blue light stimulation in SSFO-VM vs. control mice, ks-test, using the mean of each mouse (*n* = 4 mice/group). **c** CDF plots of manually scored duration of the first wake bout after stimulation during NREM and REM sleep. *p < 0.01, **p < 0.001; blue light stimulation vs. yellow light mock stimulation in SSFO-VM, paired *t*-test using mean of each mouse, blue light stimulation in SSFO-VM vs. in control mice, unpaired *t*-test (*n* = 4 mice/group)

VM activation in NREM sleep awoke the mice, but they returned to sleep quickly, consistent with the fast kinetics of C1V1 inactivation (Supplementary Fig. 7C). Next, we employed another channelrhodopsin variant, the stable step function opsin (SSFO), to test if prolonged VM activation is able to maintain wakefulness. Upon blue light stimulation, SSFO depolarizes neurons by introducing a steady inward cationic current with a minute-scale open channel state, and thus blue light stimulation for 30 s can lead to over 30 min of activation^26^. As with C1V1, SSFO-mediated VM activation quickly and reliably awakened mice from NREM sleep (Fig. 3a, b; Supplementary Fig. 10; *n* = 4 mice). Moreover, mice stayed awake for minutes after only 5 s of blue light stimulation (Fig. 3a, c), showing that persistent activation of VM results in sustained wakefulness. In SSFO-VM mice, we performed mock light stimulation using yellow light (594 nm), since SSFO is not responsive to this wavelength, and found no changes in the latency to arousal or in the length of wake bouts (Fig. 3b, c; Supplementary Fig. 11A). There were no changes also when blue light stimulation was delivered to control mice (EYFP-VM; Fig. 3b, c), demonstrating that rapidly induced prolonged wakefulness is caused specifically by VM activation. In SSFO-VM mice, the same blue light stimulation that was effective in NREM sleep did not cause behavioral arousal when delivered during REM sleep (Fig. 3b; Supplementary Fig. 10B, Supplementary Fig. 11A) consistent with the results obtained in C1V1-VM mice. Interestingly, however, SSFO-VM mice stayed awake much longer after REM sleep with blue light stimulation than after REM sleep with yellow light stimulation, despite similar latencies in awakenings in the two conditions (Fig. 3b, c). Thus, light stimulation delivered during REM sleep did activate VM to some extent, as suggested by overall longer wake bouts, but not enough to actually wake up the mice. Mock stimulation (yellow light) to SSFO-VM mice during REM sleep, as well as yellow light stimulation in C1V1-VM mice, and blue light stimulation in EYFP-VM mice did not affect latency to arousal, post stimulation wake bout length (Fig. 3c).

### Optogenetic activation of ventral posteromedial nucleus does not induce arousal

**Fig. 4:**
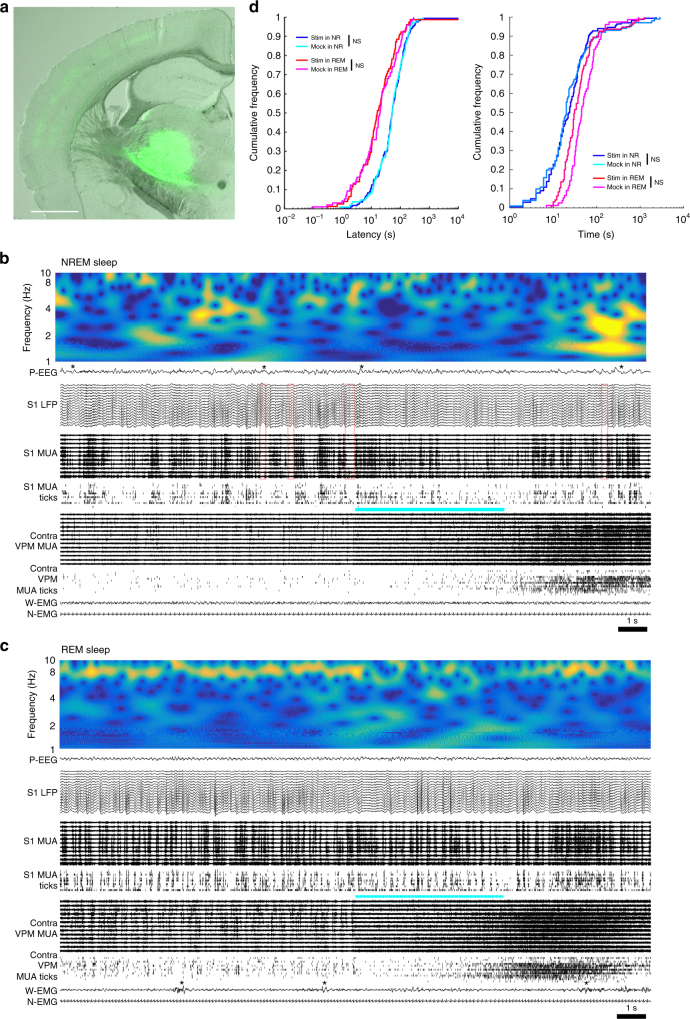
Optogenetic activation of VPM neurons does not affect vigilance states. **a** Coronal section of the mouse brain (Bregma −1.6 A/P) showing a representative example of virus expression in VPM (AAV2-Cre and AAV5-DIO-SSFO). Cortical projection is strongest in the middle layer. Scale bar = 1 mm. **b** Example of VPM optogenetic stimulation in NREM sleep, showing the 10 s before and after laser onset. Red open squares identify OFF periods. The blue bar indicates when the light is on. The 5-s pulse gradually increases VPM unit firing, with the effect becoming even stronger after light off (VPM MUA and ticks, thresholded spikes). EEG and LFP show lower amplitude at the time of stimulation, but despite strong tonic VPM firing slow waves (*) quickly reappear, associated with increased SWA (top panel). Both EMG signals are unaffected. **c** Example of VPM stimulation in REM sleep. * indicates a sporadic whisker movement. **d** CDF plots of automatically detected latency to awakening after VPM stimulation (left) and manually scored duration of wake bouts immediately after stimulation in SSFO-VPM mice (right). NS; *p* > 0.05, paired *t*-test using mean of each mouse (*n* = 4 mice)

Next, we asked if the other major type of thalamic cells, the core cells, are able to induce arousal from sleep. To target core cells, Cre-dependent SSFO and AAV-Cre were mixed and injected into the ventral posteromedial nucleus (VPM) of wild-type B6 mice (*n* = 4 mice). VPM receives sensory inputs from the whiskers and as expected, projections from VPM were seen mainly in the middle and deep layers of somatosensory cortex (Fig. 4a). Optical stimulation of VPM induced prolonged activation of its neurons during both NREM sleep and REM sleep (Fig. 4b, c) and transiently affected the EEG, but the typical EEG patterns of NREM and REM sleep were quickly restored, and marked changes in the EMG signals were never observed. Blue light stimulation did not shorten the latency to arousal from either NREM or REM sleep compared to mock stimulation with yellow light, nor did it extend wake bout duration after the stimulation (Fig. 4d). Thus, contrary to matrix cells, the activation of core cells did not lead to arousal, neither from NREM sleep nor from REM sleep.

### Within-state Granger causality analysis

**Fig. 5:**
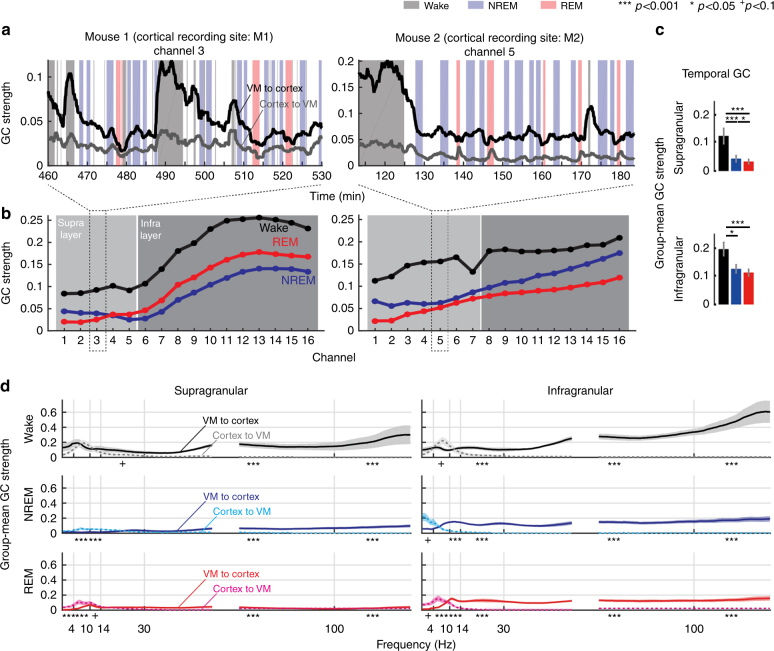
Vigilance state-dependent Granger Causality (GC) between VM and motor cortex. **a** Raw traces of temporal GC from M1 or M2 to VM (gray line) and VM to M1 or M2 (black line). **b** Temporal GC strengths across all cortical channels, in the same mice shown in **a**. Light or dark shaded area indicates the range of supra- or infra-granular layers, respectively. **c** Group-mean temporal GC strengths in the supra- or infra-granular layer channels. **d** Directed causal influence between VM and cortex through distinct frequency channels. In all columns, wake (top), NREM sleep (middle), and REM sleep (bottom). All error bars show standard errors of GC strengths (*n* = 5 mice)

Next, we measured Granger causality (GC) between VM and cortical activity in each behavioral state. We used the LFPs of consolidated periods of wake, NREM and REM sleep and quantified first the temporal GC, i.e., the mean GC strength across all frequencies (0.5–100 Hz), for one representative VM channel and all 16 cortical channels. In both wake and sleep, GC strength was higher from VM to cortex than from cortex to VM, supporting the idea that VM can influence the cortex but not vice versa (Fig. 5a). We also found that GC strength from VM to cortex varied depending on vigilance state, being always higher during wake than during NREM sleep and REM sleep, in both M1 and M2, and across all cortical channels (Fig. 5b). Overall, when both areas were considered together, GC strength was roughly twice as high in wake than in NREM in infragranular layers, and 3–4 fold higher in supragranular layers (Fig. 5c). During NREM sleep VM neurons fire at much lower rate than during wake (Fig. 1b), which may explain why their influence on cortex is weaker. After considering the major frequency bands separately, we also found that low and high frequencies behaved differently: delta (SWA) and theta frequencies showed a trend toward stronger GC from cortex to VM across all states, and this effect became significant during sleep (Fig. 5d). We also conducted additional analyses to determine whether VM spike activity can drive, or only modulate, cortical activity. First, we calculated cross-correlograms of thalamocortical neuron pairs by using thalamic neurons as references, and found little evidence for time locking between cortical and VM spiking (Supplementary Table 1). On the other hand, GC analysis based on spike trains between VM and cortical neurons revealed that all cortical neurons in all mice receive significant causal influences from VM activity (Supplementary Fig. 12). Moreover, pairwise spike count correlation (SCC) analysis found that cortical SCC, a measure of functional connectivity, increased when the influence of VM activity on cortical neurons was removed (Supplementary Fig. 13, and see Methods for details). Together, these results suggest that VM activity modulates cortical activity, and its overall effect is to uncouple and desynchronize cortical local circuits, in line with the general role of activating systems.

### Optogenetic activation of VM induces arousal from anesthesia

We also studied the effects of VM activation during anesthesia. A subset of mice (*n* = 6, two C1V1-VM, four SSFO-VM) were anesthetized with sevoflurane inside a gas anesthesia chamber (5% for induction) and injected with dexmedetomidine (50–70 μg/kg). In pilot studies, we found that this combination of drugs reliably induces a stable pattern of slow waves. After the mice lost their righting reflex and laid on their side, they were exposed to constant concentration of sevoflurane (1–1.2%) throughout the experiment. After at least 10 min of a stable state with cortical slow waves, light stimulation was delivered, resulting in rapid cortical EEG activation in all six animals (Supplementary Fig. 14, Supplementary Fig. 15). In five mice, cortical activation was followed by continuous limb movements (starting 30, 33, 82, 90, 120 s after light onset), and recovery of the righting reflex occurred in four animals (54, 141, 146, 275 s after light onset). We also assessed the effects of VM activation with an automatic method to quantify motor activity (whisker EMG), using the 10 s before stimulation onset as baseline EMG values. In all mice, including the one in which overt limb movements were not seen, the established EMG threshold (>mean + 10 std of baseline) was reached faster after VM activation relative to before (Supplementary Fig. 16A; *n* = 6 mice, *p < 0.05, paired *t*-test), indicating that VM optogenetic stimulation reliably increases muscle tone under anesthesia. VM optogenetic activation also affected the cortical EEG power, decreasing SWA and theta power, shifting the SWA peak to faster frequencies, and increasing gamma power (Supplementary Fig. 16B). Overall, these results suggest that VM-induced arousals from NREM sleep and slow wave anesthesia are qualitatively similar. Perhaps not surprisingly, however, awakenings from anesthesia took longer, even though stronger stimulation was applied. Note that in REM sleep, stimulation as strong as those used in the anesthesia experiments failed to induce arousal as quickly and reliably as in NREM. Taken together, these experiments show that the activation of VM matrix cells elicits behavioral arousal from slow wave states, in all cases from NREM sleep and in most cases under slow wave anesthesia, and can promote sustained wakefulness after awakening. By contrast when given during REM sleep, the same stimulation only subtly affects the EEG power spectrum and never leads to behavioral arousal.

### Chemogenetic inactivation of VM increases NREM sleep duration

Next, we examined the effects of VM inactivation on vigilance states. We chose a chemogenetic approach because it allows for sustained inhibition of VM cells. To control for its possible sedative effects, the selective ligand clozapine-*N*-oxide (CNO) was injected in Calb1-Cre mice expressing either the inhibitory receptor hM4Di (M4 + , *n* = 5 mice) or the non-hM4Di AAV mCherry (controls, *n* = 4 mice). CNO was injected just before the beginning of the dark phase, when mice are mostly awake. As expected, both M4+ and control mice stayed mostly awake after receiving vehicle (DMSO) injection, and there were no significant differences in wake, NREM sleep or REM sleep duration between the two groups (Supplementary Fig. 17A, B; % time spent in wake, first 6 h after injection, mean ± sd, controls: 68.1 ± 9.7%; M4 mice: 59.9 ± 7.3%; *p* > 0.05; see Methods LME model for details). By contrast, after CNO injection M4+ mice spent less time awake and more time asleep relative to controls (Supplementary Fig. 17A, B), with significant increases in both NREM and REM sleep duration (first 6 h after injection, mean ± sd, controls vs. M4 + mice, % of wake 70.9 ± 10.3% vs. 53.5 ± 6.9%, z = −2.85, *p* = 0.0074, 17.7% decrease; Methods LME model for details).

## Discussion

We have shown here that matrix cells concentrated in the “non-specific” thalamic nucleus VM powerfully control cortical activity and promote arousal, whereas the stimulation of core cells in the “specific” thalamic nucleus VPM has none of these effects. Because of its characteristically diffuse and superficial projection pattern, VM had long been implicated in the induction of the recruiting response^20,27^, a rhythmic, synchronous and widespread response elicited in the cortex by low frequency stimulation of many areas in the medial thalamus^16^. More generally, VM had long been considered the rostral extension of the upper reticular core of the brainstem, sharing an ascending activating role with some intralaminar thalamic nuclei including the centrolateral–paracentral complex^1,27^. Until now, however, direct evidence for a role of VM in arousal was limited. Electrolytic lesion studies are not specific^25^, and pharmacological studies had focused on the motor effects of GABAergic drugs infused in VM, based on its strong connections with the substantia nigra. The overall result was that bilateral injections of muscimol caused catalepsy (frozen behavior) without rigidity and without loss of righting reflex, while bicuculline infusion caused locomotor hyperactivity^28,29^, but again these manipulations were not specific. Here we show that firing in VM neurons on average precedes cortical activation, and their specific stimulation awakens mice from NREM sleep and anesthesia, providing direct evidence for their role in arousal. Notably, VM receives modest input from the midbrain reticular formation and sends only a minor output to it^27^. Thus, it is unlikely that laser pulses directed to VM caused arousal indirectly via stimulation of midbrain reticular neurons. We also show, using GC analysis, that VM effects on cortex are stronger during wake than during NREM and REM sleep, even though in REM sleep VM cells fire at wake-like levels. Moreover, different analyses of spike trains between VM and cortex showed that the VM has modulatory, rather than driving, effects on cortical activity. Altogether, the present results establish a specific causal role for matrix cells in mediating the effects on cortical arousal classically attributed to “non-specific” thalamic nuclei, consistent with the recently established role of the thalamus in promoting functional cortical connectivity^30,31^. The factors affecting changes in the firing and excitability of matrix cells across behavioral states remain to be determined.

Previous studies had examined the role in seizure induction of the centromedial nucleus (CM)—another classic “non-specific” nucleus of the thalamus^32,33^. Infusion of GABA agonists into CM depresses arousal and facilitates the induction of seizures by other drugs, suggesting that GABAergic transmission in CM indirectly controls seizures by regulating the excitability of other structures^34^. CM infusion of either nicotine or an antibody against the voltage-dependent potassium channel Kv1.2 led to the recovery of the righting reflex in rats anesthetized with desflurane (3.6%) or sevoflurane (1.2–1.4%), indicating that CM stimulation can increase arousal^32,33^. In those studies EEG and unit recordings were not performed, hence behavioral arousal could not be linked to specific changes in thalamocortical activity. Infusions centered on CM were effective in 75% of cases, while injections located more dorsally or ventrally caused arousal only in 17% of cases. When it occurred, however, awakening was slow, happening on average 5 min (nicotine) or 3 min (anti-Kv1.2) after the infusion. Moreover, the injection volume was relatively large (0.5 µl), raising the possibility that the primary effective site was outside CM itself. Also, arousal lasted 1 min after nicotine or 6–7 min after anti-Kv1.2 and the infusion frequently caused seizures. The connectivity of CM also suggests that this nucleus is not likely to mediate widespread changes in cortical excitability because CM contains very few cells projecting to L1 compared to VM, and each of these cells tends to project to a single cortical area rather than having branching axons that reach multiple areas like VM neurons^17–19^. In line with this interpretation, a recent study showed that chemogenetic activation of a large group of glutamatergic neurons, encompassing several midline and intralaminar nuclei including CM, increased high gamma activity across all behavioral states, but did not affect wake duration^35^.

An intriguing finding was that optogenetic activation of VM was unable to awaken the mice from REM sleep. Such a striking difference in the ability to induce arousal from NREM and REM sleep with manipulations that affect the thalamus was reported before, but the underlying mechanisms have remained obscure. Experiments in diencephalic cats, which lack cortex and striatum but have a relatively preserved ventrobasal and midline thalamus, showed that olfactory or acoustic stimulation could induce arousal from NREM sleep but not from REM sleep^36^, while in athalamic cats even the strongest stimulus was unable to induce arousal from either phase of sleep^6^. A recent study found that acute inhibition of the inhibitory reticular thalamic nucleus also induces arousal from NREM sleep and slow wave anesthesia in a powerful and specific manner, while increasing the activity of reticular cells stabilized NREM sleep^37^. Remarkably, the same manipulation of the reticular thalamic nucleus also did not awake animals from REM sleep, similar to our findings with VM stimulation. Optogenetic activation of the cholinergic system also promotes arousal when it occurs during NREM sleep but not during REM sleep, which is either unaffected or prolonged by the laser pulses^38–41^. Finally, optogenetic activation of the dopaminergic ventral tegmental area neurons causes an almost immediate transition from NREM sleep to wake and is only partially effective in REM sleep, decreasing its duration but not causing immediate arousal^42^. In stark contrast, optogenetic stimulation of the noradrenergic system of the locus coeruleus^43^ and of the hypocretin/orexin system of the lateral hypothalamus^44^ induces arousal both from NREM sleep and from REM sleep. Notably, noradrenergic and orexinergic cells are silent in REM sleep^45–47^. By contrast, cholinergic neurons^48,49^ and dopaminergic neurons in the ventral tegmental area^42^ are even more active in REM sleep than in wake. As shown in this study, VM cells fire almost as much in REM sleep as in wake. In cats, VM receives projections from the reticular formation in the pons and medulla, and medullar magnocellular neurons may be especially important for the tonic EEG activation during REM sleep, when they fire most strongly^50,51^. A possible interpretation of these various findings is that the experimental activation of neuronal groups that are already spontaneously active in REM sleep, including VM cells, may not cause arousal because they are part of the network that sustains REM sleep. Hence, their stimulation will rather maintain this phase of sleep than force a state transition. Another possibility is that failure to arouse from REM sleep is linked to the high cholinergic tone of this phase of sleep, coupled with the lack of noradrenaline, but future experiments are needed to test this hypothesis. In short, we demonstrate that matrix cells in VM are capable of causing behavioral arousal and diffuse cortical activation during NREM sleep and slow wave anesthesia, but the reasons why they fail to do so during REM sleep remain unclear, one of the many paradoxes of REM sleep.

## Methods

### Animals

Adult (at least 9 week old; body weight 23–32 g) male mice were used, including Calb1-Cre (B6.Cg-Calb1tm1.1(folA/cre)Hze/J mice (Stock N, #023531) and C57BL/6 wild-type mice, both obtained from Jackson Laboratory. All animal surgical procedures were used with approval from the National Institutes of Health Guide for the Care and Use of Laboratory Animals. Facilities were reviewed and approved by the IACUC of the University of Wisconsin-Madison, and were inspected and accredited by AAALAC.

### Validation of Calb1-Cre line and estimate of virus infection rate

The Calb1-Cre line that we used expresses a trimethoprim-inducible EGFP/Cre fusion gene under the control of endogenous Calbindin 1 promoter/enhancer elements and after induction, it is expected to show Cre recombinase activity in scattered cells of the cortex, hippocampus, cerebellum, and striatum, as well as in specific thalamic and hypothalamic cell groups (Allen Mouse Brain Atlas, Experiment 326746663, 326746879). To confirm the expected distribution of Cre recombinase activity in VM and estimate the virus infection rate, we injected Cre-dependent EYFP virus [AAV5-EF1a-DIO-EYFP] and examined the expression of Cre-dependent EYFP and endogenous Calb1 in the transgenic line. Calb1-Cre mice were fitted to a stereotactic frame under isoflurane anesthesia (2% induction; 1–1.5% maintenance). Using sterile technique, a midline incision was made to expose the skull and after cleaning with saline and hydrogen peroxide, two small burr holes were made in the skull using a dental drill. Cre-dependent EYFP was injected bilaterally in VM, slightly dorsal and lateral relative to the center of the nucleus to avoid damage (mm from Bregma according to ref. ^52^ −1.46 A/P, +1.1 M/L, −4.2 D/V; 0.1 µL/min, 0.5 µL total/side). Trimethoprim was administered for three consecutive days to induce stable expression of Cre protein (i.p., 0.25 g/kg, starting at least 4 days after virus injection). At least 4 weeks after the induction of the Cre protein mice were transcardially perfused under deep anesthesia (3% isoflurane in oxygen) with 4% paraformaldehyde (PFA) with a 24-h post fix in PFA. Brains were subsequently sectioned with a vibratome (Leica) into 50 µm thick sections. Coronal sections containing VM were taken from three mice and stained for endogenous Calb1 as described below (see Histology; cortical projections from thalamus), using an anti-Calb1 antibody (mouse monoclonal anti-Calbindin D-28k IgG, SWANT and Goat anti mouse IgG Alexa fluor 568, Thermo Fisher, A11004). In each section, 512 × 512 pixel images were acquired in left and right VM with a confocal microscope (Olympus BX61W1, ×40) using the red and green channels. Calb1-positive cells and EYFP-positive cells were counted independently in each channel and then compared to assess the degree of co-labeling. Coronal sections around +1.7 A/P mm from Bregma were stained with the nuclear marker BOBO-3 (Thermo Fisher, B3586) to visualize cortical layer boundaries.

### Virus injections

For optogenetic experiments, using the same technique described above, C1V1 [AAV5-EF1a-DIO-C1V1(E122T/E162T)-TS-EYFP] or SSFO [AAV5-EF1a-DIO-hChR2(C128S/D156A)-EYFP] was injected bilaterally in the VM of Calb1-Cre mice (*n* = 4 mice for C1V1, *n* = 6 mice for SSFO). For optogenetic experiments in VPM, Cre [AAV2-PKG-Cre] and SSFO were injected bilaterally in the VPM of B6 mice (−1.7 A/P, +1.5 M/L, −3.75 D/V; 0.1 µL/min, 0.5 µL total/side). Mice were monitored daily for 7 days following surgery to ensure normal recovery. In Calb1-Cre mice, Trimethoprim was administered for three consecutive days to induce stable Cre expression (i.p., 0.25 g/kg, starting at least 4 days after virus injection). AAVs for optogenetic experiments were purchased from the University of North Carolina (UNC) Vector Core (titer range 2.0 × 10^12^–8.0 × 10^12^ GC/ml), under an agreement with Dr. Karl Deisseroth. For chemogenetic experiments, DREADD M4 [AAV8-hSyn-DIO-hM4D(Gi)] or mCherry [AAV8-hSyn-DIO-mCherry] was injected bilaterally in the VM of Calb1-Cre mice (*n* = 4–5 mice/group; University of North Carolina, UNC Vector Core RRID:SCR_002448; based on previous work by Dr. Bryan Roth).

### Histology: determination of recording site and depth

Mice were transcardially perfused under deep anesthesia (3% isoflurane in oxygen) with 4% PFA with a 24-h post fix in PFA. Brains were subsequently sectioned with a vibratome (Leica) in 50 µm thick sections. In mice used for electrophysiology, the recording location of both cortical and thalamic probes was confirmed histologically. Mice in which the tip of the thalamic probe was outside VM were excluded. When the tip was inside VM, power spectrum analysis was performed using all 16 channels of the probe, after confirming that the channels gave consistent results. The recording depth of cortical probes was estimated functionally in vivo by looking at changes in LFP gamma power (40–100 Hz) across cortical layers, since previous studies showed that the gamma peak occurs in L4 in sensory cortex and at the L3/L5A border in motor cortex^53^. To correct for differences in implant depth across mice, we set the depth of the channel with the gamma peak as 0 and then assigned each channel a depth relative to 0.

### Histology: cortical projections from thalamus

In mice used for optogenetic experiments, a set of coronal sections spaced ~0.5 mm apart and spanning from +3 to –3 mm from Bregma were used for immunostaining. Sections were washed with PBS (pH 7.4), treated with 0. 3% Triton X-100 in PBS followed by a blocking solution (5% NGS, 1% BSA and 0.3% Triton X-100) for 1 h, and incubated overnight at 4 °C in the blocking solution containing rabbit anti-GFP antibody (1:500, Thermo Fisher, A21311). Sections were then washed with PBS, incubated with 5% NGS PBS containing the secondary antibody (1:500, Goat anti-Rabbit IgG Alexa Fluor 488, Thermo Fisher, A11008) for 2 h at room temperature. Images from all cortical areas were acquired using both the bright-field and the green fluorescent channel (Leica DMR/EC3 system). Based on the bright-field image, major cortical areas were identified and regions of interest (ROIs) were drawn to span the different layers of each cortical area. The ROIs were then applied to green fluorescent channel images and the EYFP signal intensity was measured.

### Electrodes implant

At least 2 weeks after the AAV injections, mice were anesthetized with isoflurane (2% induction; 1–1.5% maintenance) and implanted with electrodes to assess brain and muscular activity. The EEG was recorded by fixing gold screws in burr holes (0.7 mm) above frontal and/or parietal cortex, with a screw above the cerebellum serving as reference. Anchor screws were also placed above the cerebellum and olfactory bulbs. To record cortical activity, craniotomies were performed at target cortical areas, ensuring that the dura remained intact, followed by insertion of laminar silicon probes (NeuroNexus, A1x16, 50 or 100 µm site spacing) perpendicular to the target cortical regions. The D/V dimension was adjusted during surgery to ensure that the top contact was placed at the surface of cortex. For thalamic recordings, silicon probes (NeuroNexus, A1x16 Poly2, 16 contacts arranged in two columns spaced between 0.068 mm and 0.443 mm from the tip) were used (VM; −1.46 A/P, + 0.9 M/L, −4.3 D/V; VPM; −1.9 A/P, +1.5 M/L, −3.75 D/V). While the virus was injected bilaterally, only one thalamic probe was implanted to monitor VM activity on one side. For optogenetic stimulation, one or two optic fibers (Doric lenses, core diameter 200 µm, NA 0.22, diffuser type tip) was implanted dorsal to VM or VPM. Two-component silicon (QuikSil) was applied to craniotomy, probe, and optic fiber, followed by dental acrylic (Fusio) to secure the array. To record muscle activity a pair of custom-made electromyogram (EMG) cables were inserted bilaterally into the dorsal neck musculature and in the vibrissal musculature.

### Chronic sleep recordings

Implanted mice were singly housed in transparent Plexiglas cages (36.5 × 25 × 46 cm) for the duration of the experiment (light/dark 12:12, light on at 8:00 a.m., 23 ± 1 °C; food and water available ad libitum). After at least 1 week of recovery from surgery, chronic 24-h recordings started at the beginning of the light phase. Data acquisition was performed with the Multichannel Neurophysiology Recording and Stimulation System (Tucker-Davis Technologies Inc., TDT), with spike data being accumulated continuously across the 24-h cycle. LFPs (256.99 Hz, 0.1–100 Hz), multi-unit activity (MUA; 24.4 kHz, 300–5000 Hz), surface EEG (256.99 Hz, 0.1–100 Hz), and EMG (256.99 Hz, 10–100 Hz) were acquired using a PZ amplifier and RZ2 system (TDT). LFPs, EEGs, and EMGs were then resampled to 256 Hz except for spike-locked LFP analysis. Voltage thresholds for MUA were determined by visual inspection. Both time stamps and waveforms (46 peri-threshold crossing samples) were attained for each supra-threshold event.

### Optogenetic stimulation

In all experiments laser pulses were triggered remotely to avoid interfering with the spontaneous behavior of the mice, and the intensity of laser stimulation was tested in pilot experiments. Real-time brain activity and EMG signal were monitored remotely and laser stimulation was delivered after at least 15 s of stable NREM sleep or REM sleep. After laser onset, time to awaken and time to resume consolidated sleep (at least 20 s) were calculated manually based on brain activity and EMG signal. In C1V1-VM mice, yellow light (594 nm, up to 10 s) was used for stimulation. Laser pulses were delivered starting 3 h after light onset until the end of the light phase. After one laser pulse, the next one was delivered after at least 10 min of consolidated sleep, since pilot experiments showed that frequent stimulations led to a decrease in response. Two patterns of yellow light stimulation were used, and since they had similar effects, the results were pooled (25 Hz, 15 ms pulses or 0.5 Hz, 1 s pulses, both lasting up to 10 s). Negative control stimuli (laser power set at 0) were also delivered to measure the distribution of latency to awaken and latency to consolidated sleep in the absence of any laser stimulation. The same experimental design was applied to SSFO-VM mice, with a few modifications. Blue light (473 nm, up to 5 s) and yellow light (594 nm, 5 s) were used for stimulation and mock stimulation, respectively. After one laser pulse, the next one was delivered after at least 30 min of consolidated sleep, since SSFO stimulation caused a significantly longer wake period than C1V1 stimulation. The light intensity required to induce a consistent response was variable across both C1V1-VM and SSFO-VM mice. In EYFP-VM (control) mice and in SSFO-VPM mice an optimal laser power could not be established in pilot experiments; thus, these animals received the maximum yellow light intensity applied to C1V1-VM animals, and multiple light intensities ranging from the lowest to the highest blue light used in SSFO-VM animals. In a subset of C1V1-VM mice and SSFO-VM mice, VM was optogenetically stimulated under anesthesia. Mice were anesthetized with 5% sevoflurane (for induction) and dexmedetomidine (50–70 µg/kg, i.p.) was administered after the loss of the righting reflex. Mice were then placed in a clear acrylic anesthesia chamber with an isothermal heating pad and exposed to 1–1.5% sevoflurane in oxygen at 1.5 l/min. Sevoflurane concentration was monitored (Criticare, Poet IQ2). Laser stimulation started after at least 10 min in which the EEG pattern was dominated by slow waves (about 1–2 Hz).

### Chemogenetic inhibition

At least 4 weeks after virus injections, Calb1-Cre mice were implanted with electrodes for chronic 24-h recordings of vigilance states, which started at least 1 week after surgery. The day of the experiment sleep and wake were monitored starting from the onset of the light period (8:00 a.m.) and mice were injected with either vehicle (1% DMSO in saline, i.p.) or ligand (clozapine-*N*-oxide; CNO; 5 mg/kg, i.p.) just before the beginning of the dark period (8:00 p.m.). Vehicle and CNO injections were counterbalanced in each mouse and spaced apart by at least 2 days. Vigilance states were manually scored by investigators blind to experimental conditions. Effects of virus type (hM4D(Gi) vs. mCherry) and injection (CNO vs. vehicle) were analyzed using Linear mixed effect (LME) models (see below). The first 20 min after the i.p. injection were excluded from the analyses to avoid confounding effects by the presence of investigators and physical stimulation. Motor tasks were performed as described before^54^ and i.p. injections (vehicle or CNO) were given 30 min before the onset of motor training.

### Sleep scoring

Signals were loaded with a Matlab script and subsequently processed into European Data Format (EDF). EEG, cortical LFP, and EMG activity were analyzed off-line by visual inspection of 4-s epochs using a graphic interface (SleepSign, Kissei Comtec) where all signals are displayed simultaneously to determine behavioral state. Sleep/wake scoring was done according to standard criteria and vigilance state could always be determined. Wake was characterized by low amplitude, high frequency EEG/LFP activity, and high EMG content. NREM sleep was characterized by high amplitude, low frequency EEG/LFP activity, and low EMG activity. In REM sleep, EEG/LFP activity was similar to wake, but accompanied by strong theta (6–10 Hz) in EEG/LFP channels and occasional high whisker EMG activity. The 12 h of the light phase and the first 2 h of the dark phase were scored to ensure proper entrainment to the light/dark cycle, but only data from the light phase were used in all electrophysiological analyses.

To measure mean firing rates for each vigilance state, the following criteria were used: wake:>80 s, excluding the first and last 12 s; NREM sleep:>80 s, excluding the first 20 s and the last 4 s (NREM to wake) or 40 s (NREM to REM); REM sleep:>40 s, excluding the first 20 s and the last 8 s. The number of epochs used for spike sorting was the same for all behavioral states, with the number of REM sleep epochs usually being the limiting factor used to match the number of epochs in wake and NREM sleep. Active and quiet wake were distinguished based on high or low EMG content, respectively, within consolidated epochs of wake. For some analyses the epochs of consolidated NREM sleep were divided into those with high spindle activity (7–14 Hz) and those with high SWA (0.5–4 Hz). Awakenings from NREM sleep were identified by using whisker EMG activity. First, all wake epochs immediately after at least four epochs of NREM sleep were selected from manually scored vigilance states. Then, whisker EMG activity (mean ± std) in the −15 to −5 s from the beginning of each wake epoch was used to calculate the baseline value during NREM sleep and to identify the awakenings, defined as the first moment in which whisker EMG activity exceeded the mean + 10 std of baseline. Transitions from NREM to REM sleep were identified based on cortical MUA. First, at least five epochs of REM sleep following at least five epochs of NREM sleep were selected and OFF periods (at least 100 ms of coordinated silence in cortical MUA) during NREM sleep were identified in the 20 s before the first REM epoch. The onset of cortical activation in REM sleep was defined as the end of the last OFF period.

The spontaneous sleep/wake activity was quantified in the baseline day without light stimulation across all the mice used in optogenetic experiments. No marked differences were observed across groups in percentage of wake, NREM sleep, and REM sleep, probability of transition from NREM to REM sleep, and length of each vigilance state bouts, regardless of genetic background, virus type, and location of virus injection site (Supplementary Fig. 1).

### Electrophysiological data analysis and spike sorting

All analyses were performed using Matlab scripts. For epoch selection for spike sorting, a fast Fourier transform was performed on the 4-s epochs. For cortical LFP power analysis, a fast Fourier transform was performed using 10-s epochs before and after the onset of each laser pulse, using all cortical LFP channels. Results were averaged within each vigilance state in each mouse, and collapsed across mice using the relative depth to the gamma power peak. Specifically, each channel was assigned a depth based on its distance from the channel showing the gamma peak, all channels at the same relative depth were pooled across mice, and then mean and standard deviation were obtained for each cortical depth in each cortical area. LFP channels were visually inspected and channels with significant movement artifacts were excluded from the analysis. Wavelet transform was performed to assess rapid changes in EEG/LFP power spectrum after laser stimulation, using the 10 s (sleep) or 20 s (anesthesia) before and after laser pulses were delivered.

To analyze the spontaneous activity in VM neurons and the optogenetically induced activity of VM-targeted cortical areas, spike sorting was performed off-line using supraparamagnetic clustering^55^. Visual inspection of clustered waveforms was carried out both to discriminate between single units (SU) and multi-unit activity (MUA), and to determine consistency in waveform shape. Unit stability was assessed by plotting both the firing rate in 4-s epochs and the waveform maximum amplitude. Clusters were only considered to be single units if ISIs <2 ms, which represent refractory violations, accounted for <0.5% of all ISIs. Other metrics inspected included an estimate of false negatives due to spikes missed based on the channel threshold and the distribution of waveform minima, as well as the Fisher’s linear discriminant, which identifies potential overlap of waveform characteristics between a pair of clusters on the same channel^56^. All spikes were sorted together regardless of vigilance states and then further analyzed. For spontaneous activity, all spikes from the selected epochs were used and mean firing rates for each vigilance state were obtained. In spike-locked LFP analysis, for each cluster either all spikes were used for each vigilance state (when the number of spikes in a given vigilance state was <2000), or the number of spikes was capped to 2000. Raw LFP data from −100 to +100 sampling points (~0.78 s, 1/256.99 s/sampling point × 200) relative to spike time were averaged.

To investigate the extent of synchronized firing between pairs of neurons, cross-correlogram analysis was performed using their spike trains. In each pair one neuron served as the reference neuron and the other as the target neuron. A 400 ms time window around each spike of the reference neuron was selected, spanning from −200 ms to +200 ms of the spike time, and segmented into 400 bins (1 ms each). For each bin the number of spikes of the target neuron was counted and used to make the histogram, and firing rates were calculated by converting number of spikes/bin into number of spikes/s. The level of synchronization in a pair of neurons was measured by testing whether the peak of the cross-correlogram was significantly higher than the mean + 5 std of baseline firing rate (−200 ms to −100 ms).

### GC and SCC analysis

GC analysis was first conducted using the LFP signals to examine causal influences between thalamus and cortex. GC is a statistical technique to infer causal influence between two signals based on the improvement in prediction: if the prediction of a cortical LFP signal is significantly improved when considering past values of the VM LFP signal in addition to those of the cortical signal, compared with considering only past values of the cortical signals, then GC analysis deduces that VM has a causal influence on cortical activity. Here, a nonparametric version of GC analysis (nGCA) was conducted with the FieldTrip toolbox^57^. The nGCA uses spectral density matrix factorization to estimate GC in the frequency domain, since the average strength across frequency has been theoretically proved to be identical to the temporal GC^58^. We estimated spectral density matrices in each individual mouse by using the Fourier transform of the LFP signals for all possible pairs of a representative VM channel and all cortical channels. Only one VM channel was used for this analysis because all thalamic signals were highly correlated to each other. A principal component (PC) analysis showed that the first PC explained at least 90% of the variance of all thalamic signals for all but one mouse. In the mice whose thalamic signals were explained by their first PCs, a channel was selected as representative when it had the heaviest weight of the PC. In the outlier mouse, the electrode was inserted across two different thalamic nuclei, and thus first and second PCs were needed to explain the signal variance. The representative channel for this mouse was selected as the one with the heaviest weight of the PC corresponding to VM. The spectral density matrix for REM sleep was obtained by using all epochs of REM sleep, since they were fewer than the epochs of wake and NREM sleep. The same matrix for wake and NREM sleep was estimated using the same number of epochs as in REM sleep, randomly selected. For the inspection of the temporal dynamics of GC, all epochs were decomposed into sets of 25 consequent epochs (100 s data) from which time-resolved spectral density matrices were obtained. Because a power line artifact was detected around 60 Hz, all GC estimations excluded that frequency range and spanned 0.5–55 Hz and 65–100 Hz.

For group statistics, all cortical channels in each mouse were pooled in two sets, one spanning the supragranular layers and another spanning the infragranular layers, and GC strengths were averaged in these channel sets. The lower border of the supragranular channels was identified as the channel showing the highest gamma band-limited power (mean power from 30 to 100 Hz), in agreement with a previous study^53^. Group-mean of temporal GC and band-limited GC strengths were compared across all vigilance states by permutation tests with 1000 randomizations. The band-limited GCs were calculated by averaging GC spectra within delta SWA (0.5–4 Hz), theta (4–10 Hz), alpha (10–14 Hz), beta (14–30 Hz), and gamma bands (low = 30–55; high = 65–100 Hz). Permutation tests were also used to determine whether the causal influence in one direction, thalamus to cortex, was stronger than in the opposite direction, cortex to thalamus.

To clarify whether VM spikes have any causal effects on cortical spikes we also calculated GC based on spike trains between VM and cortical neurons, as well as pairwise SCC analysis. SCC is a measure of functional connectivity and is defined as the correlation of spike count responses to repeated presentations of the same stimulus^59^. By regarding VM spikes as “stimuli” to cortical neurons, we calculated SCC in pairs of cortical neurons under two conditions. In the unshuffled condition cortical SSC was measured between two consecutive VM spikes (i.e., interspike interval of VM neurons), while in the shuffled condition we permuted interspike intervals of VM spike trains.

*Statistics*: Investigators were not blinded to mouse genotype, and no randomization was used in the optogenetics experiments. In pharmacogenetics experiments using DREADD M4, investigators scored vigilance states blindly. Sample size for electrophysiological experiments was determined based on past experience and pilot experiments. Statistics were calculated using Matlab software. Normality was assessed with Lilliefors test and the statistical significance between two independent groups was determined using Student’s *t*-test or Kolmogorov–Smirnov test. For the statistical significance between two related groups, the normality of the difference between the matched data points was assessed with Lilliefors test and two-sample paired *t*-test or Wilcoxon rank sum test was performed. Statistical tests and *p* values are described in the figure legends. All tests were two sided.

### LME models

For the chemogenetic experiments, statistical analysis was performed using LME models^60^. The use of LME models offers several advantages over traditional ANOVA methods for repeated measures including the ability to handle unbalanced designs and greater flexibility for post-hoc tests. The general matrix form of the LME is

$$y = X\beta + Zu + {\it{\epsilon }}$$

where,

$$u\sim N\left( {0,\,{\mathrm{\Sigma }}} \right),$$

and

$${\it{\epsilon }} \sim N\left( {0,\,I\sigma ^2} \right).$$

In these models, *y* is the response variable (e.g., wake duration), *u* are the random effects for each individual mouse (assumed to be independent and normally distributed, with mean zero and covariance matrix Σ), *β* are the fixed effects, and *X* and *Z* are design matrices that link the particular responses to *β* and *u*. The residuals *ϵ* are assumed to be independent and normally distributed with constant variance *σ*.

The set of random and fixed effects included in each model was determined using the Akaike Information Criterion (AIC). For the random effects, we looked for the minimum AIC value, but if several models had similar AIC values, took the one that included the most random effects, as omitting random effects can have negative consequences when testing the significance of fixed effects. Conversely, the fixed effects were selected by identifying the specific model that minimizes the AIC. Parameter estimation of LMEs was performed using numerical maximum likelihood estimators, implemented in R by the lmer() function of the lme4 package^61^. To test the significance of effects in the LME model, we used likelihood ratio tests (LRTs). Under the hypothesis that the experiment (e.g., CNO injection) had an effect on wake duration, we expected that there should be no differences between conditions (e.g., hM4D(Gi) vs. mCherry mice) after vehicle injection, but significant differences between groups on the CNO day. Thus, we specifically tested for the effect of the interaction between groups and drugs; if the interaction was found to be significant, we then performed post-hoc tests to confirm that there was in fact no difference after the vehicle injection and a significant difference after CNO injection. All post-hoc comparisons in the LME models were *z*-tests, performed using the glht() function of the multcomp package in R, with *p* values adjusted for multiple comparisons using the single-step method^62^.

The model for wake duration selected by AIC analysis included Group (hM4D(Gi) vs. mCherry, *n* = 4–5 male mice/group), Drug (vehicle vs. CNO injections), as well as a Group by Drug interaction. Parameter estimates for the LME model are shown in Table 1 below. Residual plots were used to assess the model assumptions of normality and constant variance. From the scatter plot of residuals vs. fitted values, we found no evidence against either of the assumptions. The LRT revealed a significant Group by Drug interaction (*χ*^2^ = 6.15, df = 1, *p* = 0.0131), and subsequent post-hoc tests found no significant differences between groups after vehicle injection (*z* = −1.002, *p* = 0.4364, 6.2% decrease) and a significant decrease in wake duration for the hM4D(Gi) group compared to the mCherry group after CNO injection (*z* = −2.85, *p* = 0.0074, 17.7% decrease).

**Table 1:** LME model: effect of chemogenetic inhibition of VM on wake duration

Random effects	Standard deviation		
Mouse (Intercept)	8.296		
Residual	4.068		
Fixed effects		Estimate	Standard error
Intercept		64.523	4.62
Group	mCherry (control, reference)	0	0
	hM4D(Gi)	−6.208	6.198
Drug	Vehicle (reference)	0	0
	CNO	7.498	2.876
Group by Drug interaction	mCherry, vehicle (reference)	0	0
	hM4D(Gi), CNO	−11.464	3.859

**Table 2:** LME model: effect of chemogenetic inhibition of VM on NREM sleep duration

Random effects	Standard deviation		
Mouse (Intercept)	8.169		
Residual	3.643		
Fixed effects		Estimate	Standard error
Intercept		33.308	4.472
Group	mCherry (control, reference)	0	0
	hM4D(Gi)	6.144	6
Drug	Vehicle (reference)	0	0
	CNO	−5.768	2.576
Group by Drug interaction	mCherry, vehicle (reference)	0	0
	hM4D(Gi), CNO	9.728	3.456

**Table 3:** LME model: the effect of chemogenetic inhibition of VM on REM sleep duration

Random effects	Standard deviation		
Mouse (Intercept)	0.3609		
Residual	0.5644		
Fixed effects		Estimate	Standard error
Intercept		2.169	0.335
Group	mCherry (control, reference)	0	0
	hM4D(Gi)	0.064	0.45
Drug	Vehicle (reference)	0	0
	CNO	−1.73	0.4
Group by Drug interaction	mCherry, vehicle (reference)	0	0
	hM4D(Gi), CNO	1.74	0.535

We also tested if there were significant changes in NREM and REM sleep durations (Tables 2 and 3). The AIC analyses selected the same model as the one selected for the wake duration. We found no evidence against assumptions of normality and constant variance of NREM and REM sleep durations. For both NREM and REM sleep durations, the LRT revealed significant Group by Drug interactions (NREM: *χ*^2^ = 5.68, df = 1, *p* = 0.0171; REM: *χ*^2^ = 7.04, df = 1, *p* = 0.008), and subsequent post-hoc tests found no significant differences between groups after vehicle injection (NREM: *z* = 1.024, *p* = 0.4142, 6.1% increase; REM: *z* = 0.143, *p* = 0.986, 0.06% increase) and a significant increase for the hM4D(Gi) group compared to the mCherry group after CNO injection (NREM: *z* = 2.645, *p* =  0.0133, 15.9% increase; REM: *z* =  4.01, *p* = 0.0001, 1.8% increase).

### Data availability

All relevant data are available from the authors upon request.

## Electronic supplementary material

Supplementary Information
