# Supplementary material for: Regulation of cortical activity and arousal by the matrix cells of the ventromedial thalamic nucleus
Source: Nat Commun. 2018 May 29;9:2100. doi: 10.1038/s41467-018-04497-x (PMC5974306; doi:10.1038/s41467-018-04497-x)
Supplement: Supplementary file 1 — Supplementary Information [file 41467_2018_4497_MOESM1_ESM.pdf]

Regulation of cortical activity and arousal by the matrix cells of the ventromedial thalamic nucleus

Sakiko Honjoh [1,2], Shuntaro Sasai [1], Shannon S Schiereck[1], Hirotaka Nagai[1], Giulio Tononi [1*] and Chiara Cirelli [1*]

[1] Department of Psychiatry, University of Wisconsin, Madison, Wisconsin, USA
[2] Present address: University of Tsukuba, International Institute for Integrative Sleep Medicine, Tsukuba, Ibaraki, Japan

Supplementary figures and table (20 pages)

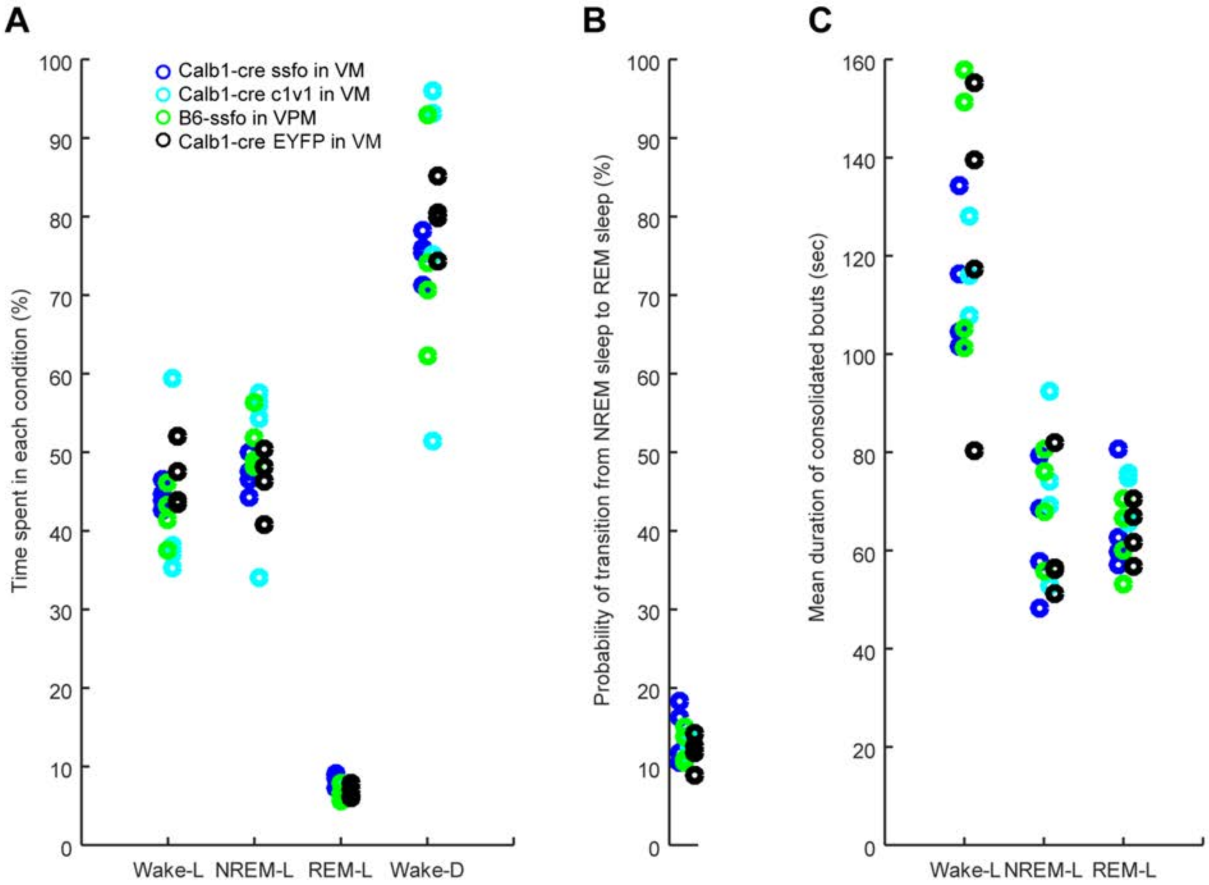

Supplementary Figure 1. Sleep/wake parameters in virus-injected mice. (A) Percentage of wake, NREM sleep, and REM sleep in the 12 hours of the light phase and wake in the first 2 hours of the dark phase. (B) Transition probability from NREM sleep to REM sleep. (C) Mean length of sleep and wake consolidated (> 12 sec) bouts. Each symbol is one mouse.

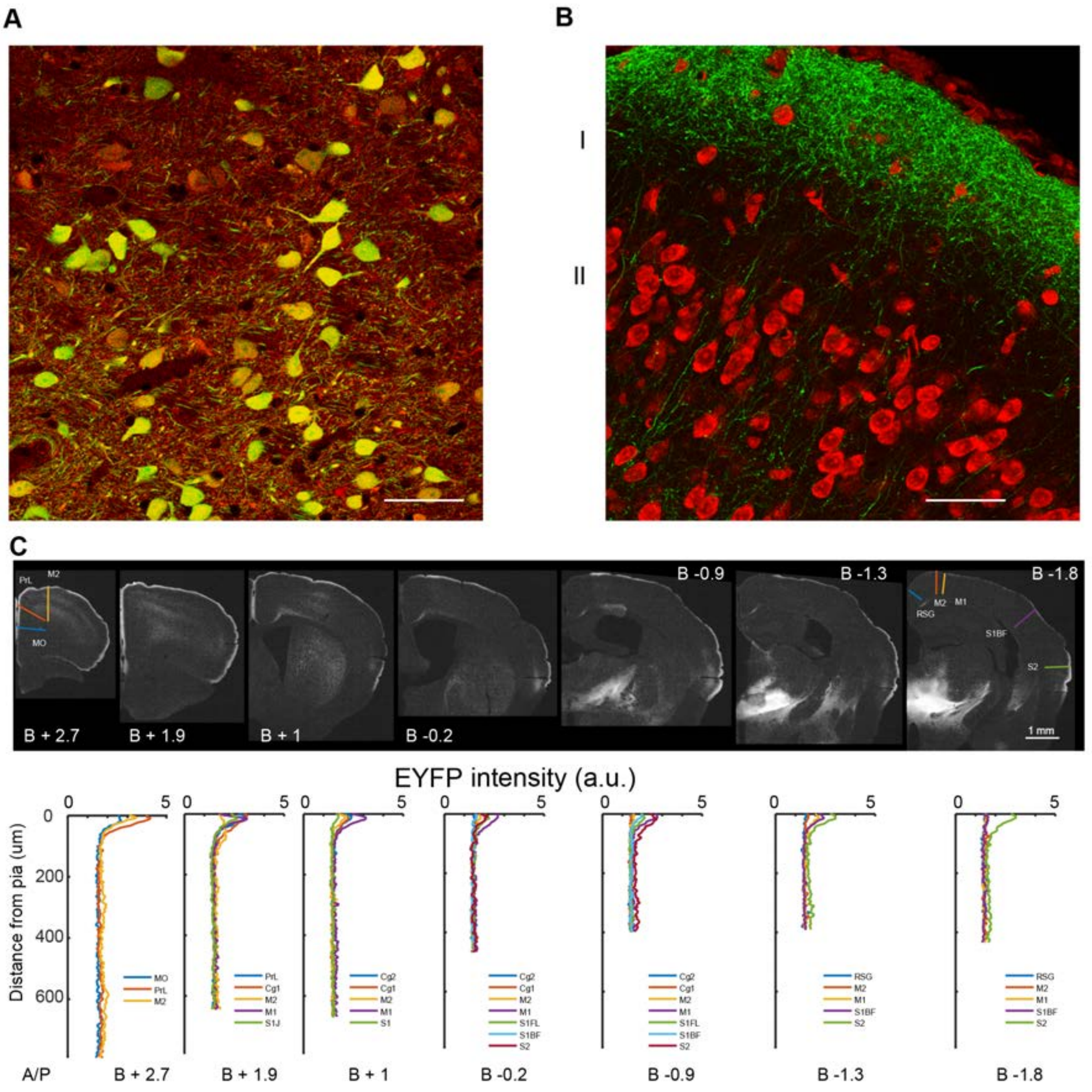

Supplementary Figure 2. Cortical projections of VM neurons. (A) Cre-dependent EYFP expression in VM neurons of Calb1-Cre mice. EYFP (green) is expressed in the 94.4% of Calb1-positive neurons (red). Scale bar, 50 µm. (B) VM axons (green) target the outer L1 of secondary motor cortex. Nucleic acid staining (red) labels all cells, delineating the boundary between L1 and L2/3. Scale bar, 50 µm. (C) VM projects widely to cortex. Top; Coronal sections at different AP levels (from Bregma +2.7 to -1.8). Cre-dependent SSFO-EYFP was injected in Calb1-Cre mice and the signal (white staining) was amplified by anti-GFP staining. Cre-dependent GFP, SSFO-EYFP, and C1V1-EYFP showed essentially the same projection patterns in Calb1-Cre mice. Lines indicate ROIs used for laminar analysis. Bottom; Quantification of the density of VM projections across layers. MO; medial orbital cortex, PrL; prelimbic cortex, M1; primary motor cortex, M2; secondary motor cortex, Cg1; cingulate cortex area1, Cg2; cingulate cortex area2, S1; primary somatosensory cortex, S1J; S1 jaw regulation, S1FL; S1 forelimb regulation, S1BF; S1 barrel field, S2; secondary somatosensory cortex, RSG; retrosplenial granular cortex.

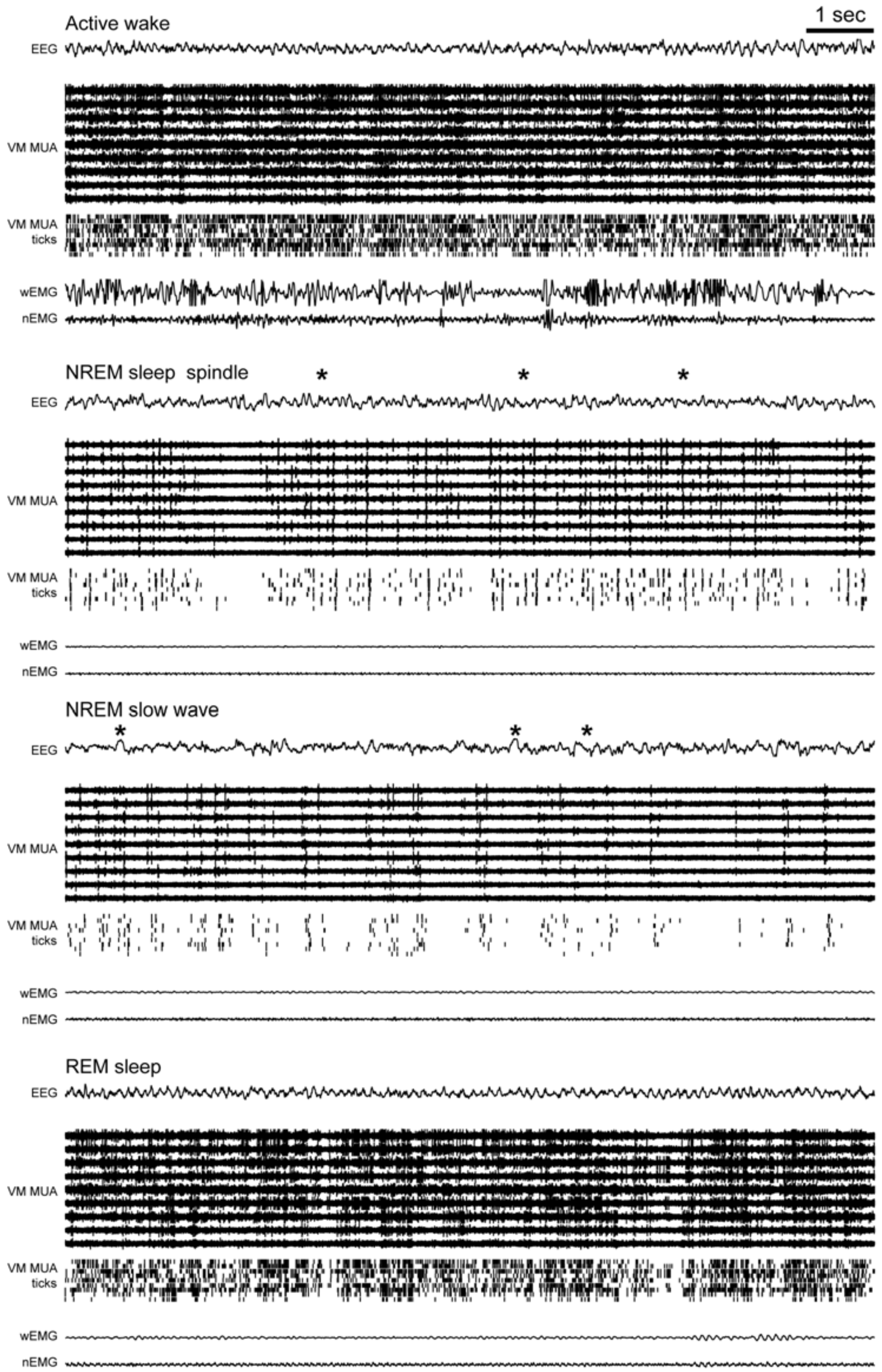

Supplementary Figure 3. VM activity across vigilance states. Raw traces of 12 seconds of parietal EEG, VM activity, and EMG from different vigilance states. VM MUA shows tonic firing in wake and REM sleep and low firing during NREM sleep, especially when the EEG is dominated by slow waves.

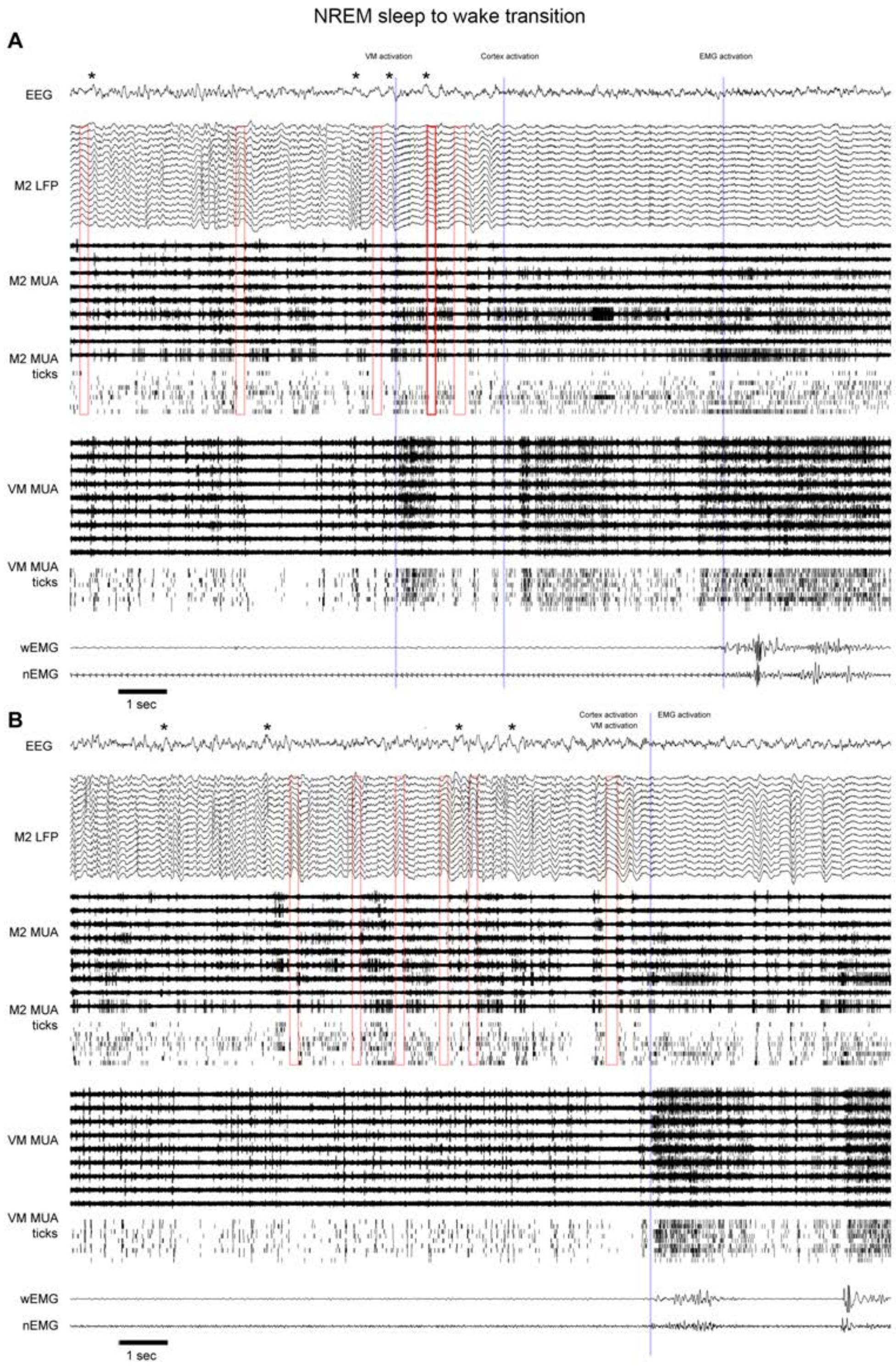

Supplementary Figure 4. Awakenings from NREM sleep. Raw traces (17 secs) of parietal EEG, M2 activity, VM activity, and EMG, with an example of VM activation preceding cortical activation (A) and simultaneous activation of VM and M2 (B). Asterisks mark individual slow waves, and pink boxes show cortical slow waves (M2 LFP) aligned with OFF periods in M2 MUA. The red box indicates a cortical OFF period during VM activation.

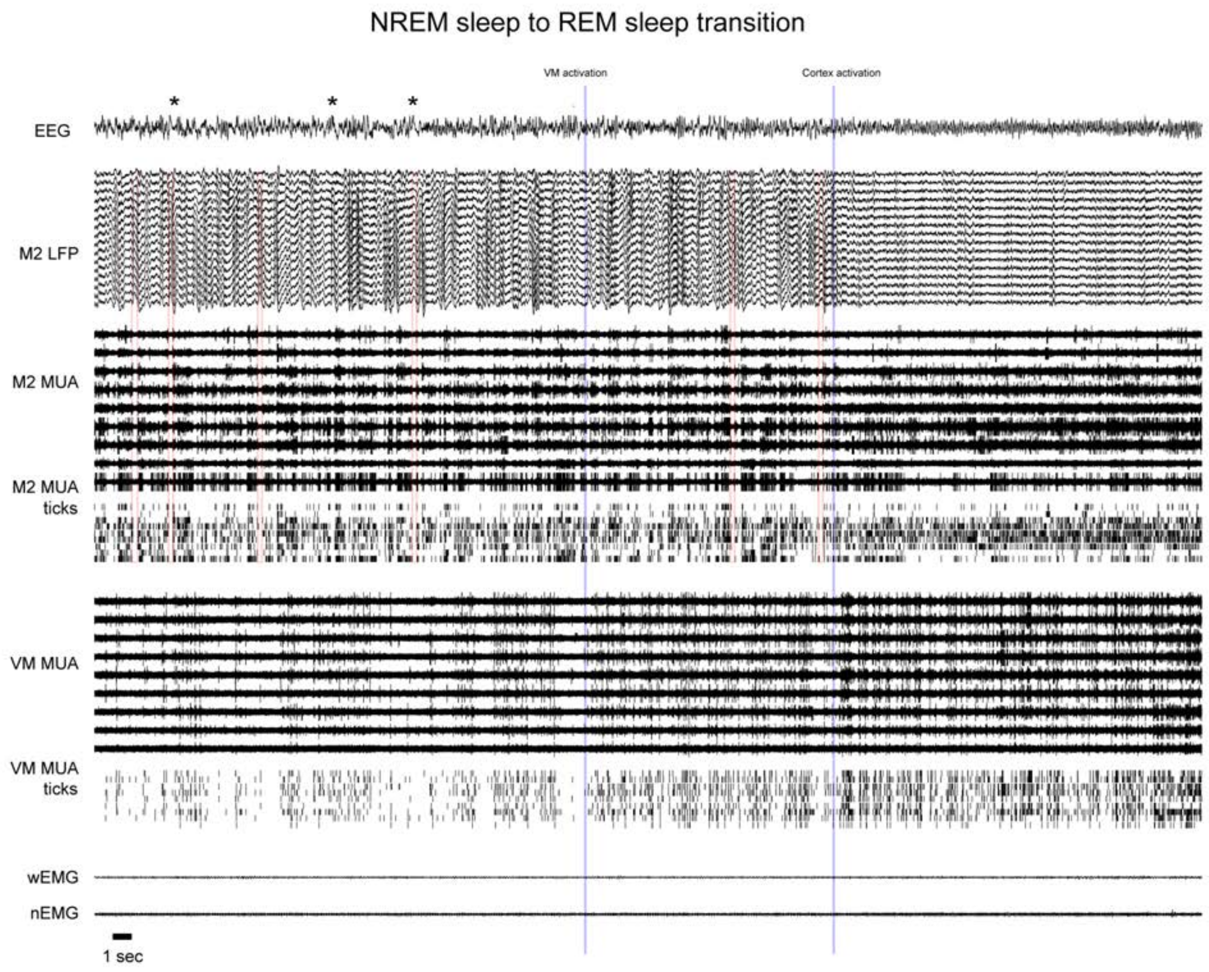

Supplementary Figure 5. Transition from NREM sleep to REM sleep. Raw traces (1 min) of parietal EEG, M2 activity, VM activity, and EMG spanning a transition from NREM sleep to REM sleep. Asterisks * mark individual EEG slow waves, pink boxes show cortical slow waves (M2 LFP) aligned with OFF periods.

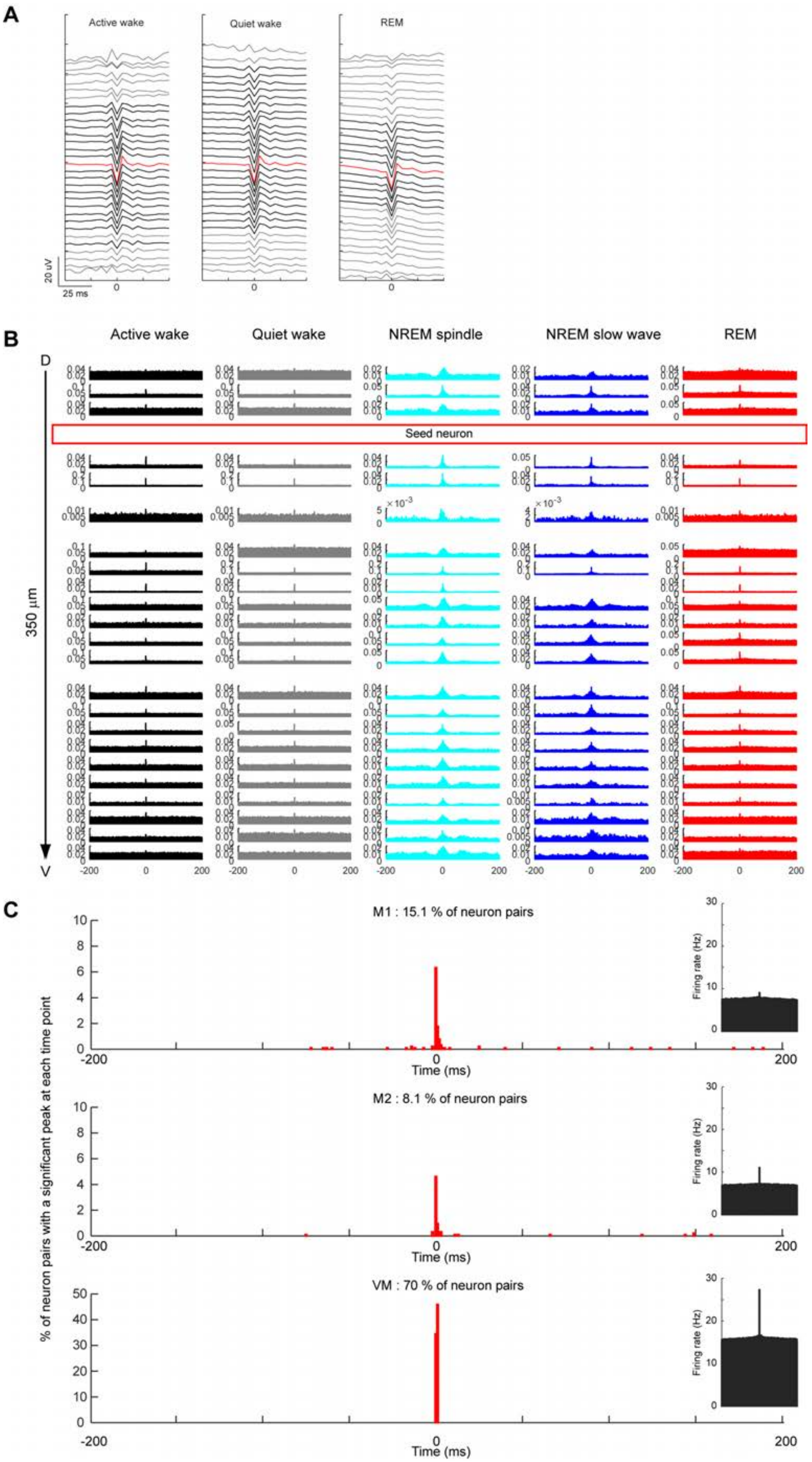
A
Active wake
Quiet wake
REM
20 uV
25 ms
0
B
Active wake
Quiet wake
NREM spindle
NREM slow wave
REM
D
Seed neuron
350 μm
V
-200
0
200
C
M1 : 15.1 % of neuron pairs
M2 : 8.1 % of neuron pairs
VM : 70 % of neuron pairs
% of neuron pairs with a significant peak at each time point
Time (ms)
Firing rate (Hz)

Supplementary Figure 6. Spike time correlation analysis. (A) Average traces of VM LFP channels locked to the firing of VM neurons (red line shows the LFP channel from which the seed neuron was recorded; for each neuron 2000 randomly chosen spikes in each vigilance state were used). Mean LFP signals locked to each VM neuron were further averaged across all sorted VM neurons (74 neurons from 10 mice). In each channel the averaged LFP signal (± approximately 390 ms from VM firing) was Z-scored. Channels with the negative peak (z-score ≤ 4 at time 0) are shown in black. (B) Example of cross-correlograms of VM neurons locked to one seed VM cell (red box) across different vigilance states. (C) Histograms showing the distribution of peak activity timing of target neurons relative to spike times of the reference neuron (peak is significant when greater than mean +5 std of the baseline, -200 to -100 ms). The Y axis shows the percentage of neuron pairs with a significant peak at each 1ms bin out of all neuron pairs examined. Insets show the average of all the cross-correlograms in each area. The percentage of neuronal pairs showing highly correlated activity is higher in VM than in M1 and M2 (15.1% in M1, 8.1% in M2, 70% in VM, VM vs. M1; p =3.8636e-04, VM vs. M2; p=1.2277e-04, t-test).

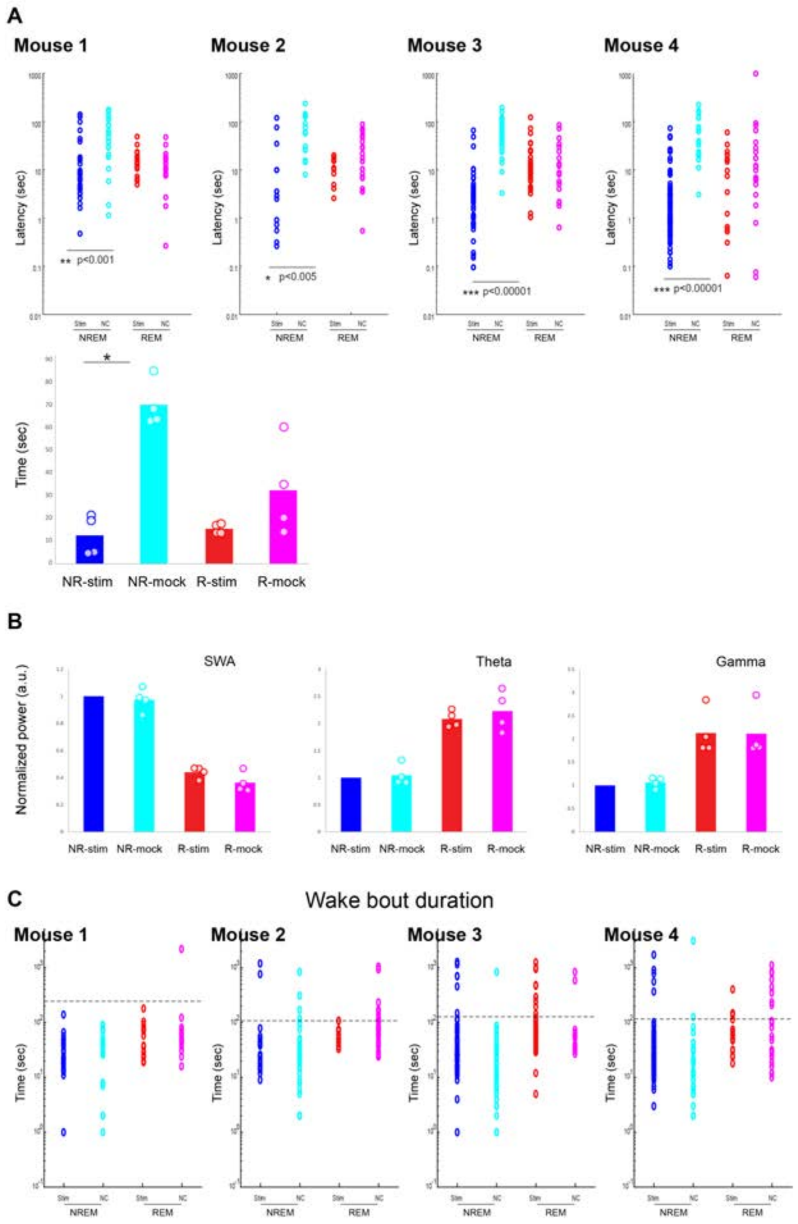

Supplementary Figure 7. Characterization of individual responses to light stimulation in C1V1 mice. Automatically-detected latency to awakening from light onset of 4 C1V1-VM mice, shown in the scatter plots for each mouse p values by t-test using all latency values from stimulation and NC are shown for NREM sleep. (A, top), and in the bar plots for the group level analysis (A, bottom). In the bar plots each dot shows the mean latency of one mouse (*; $p < 0.01$, paired t-test; n=4). NC, negative control stimulation (mock, with laser power set to 0). (B) EEG power in specific frequency bands during the last 10 secs preceding the stimulation, to show that laser pulses and mock stimulations occurred under consistent brain states. Parietal EEG power was normalized to that of NREM-stim in each frequency band (SWA; 0.5-4 Hz, theta; 6-10 Hz, gamma; 30-100 Hz). Paired t-tests (NR-stim vs. NR-mock; R-stim vs. R-mock), all p-values ≥ 0.4789. (C) Scatter plots of wake bout duration immediately after light stimulation for all pulses in the 4 C1V1-VM mice. Dotted lines show the mean length of wake bouts of each mouse.

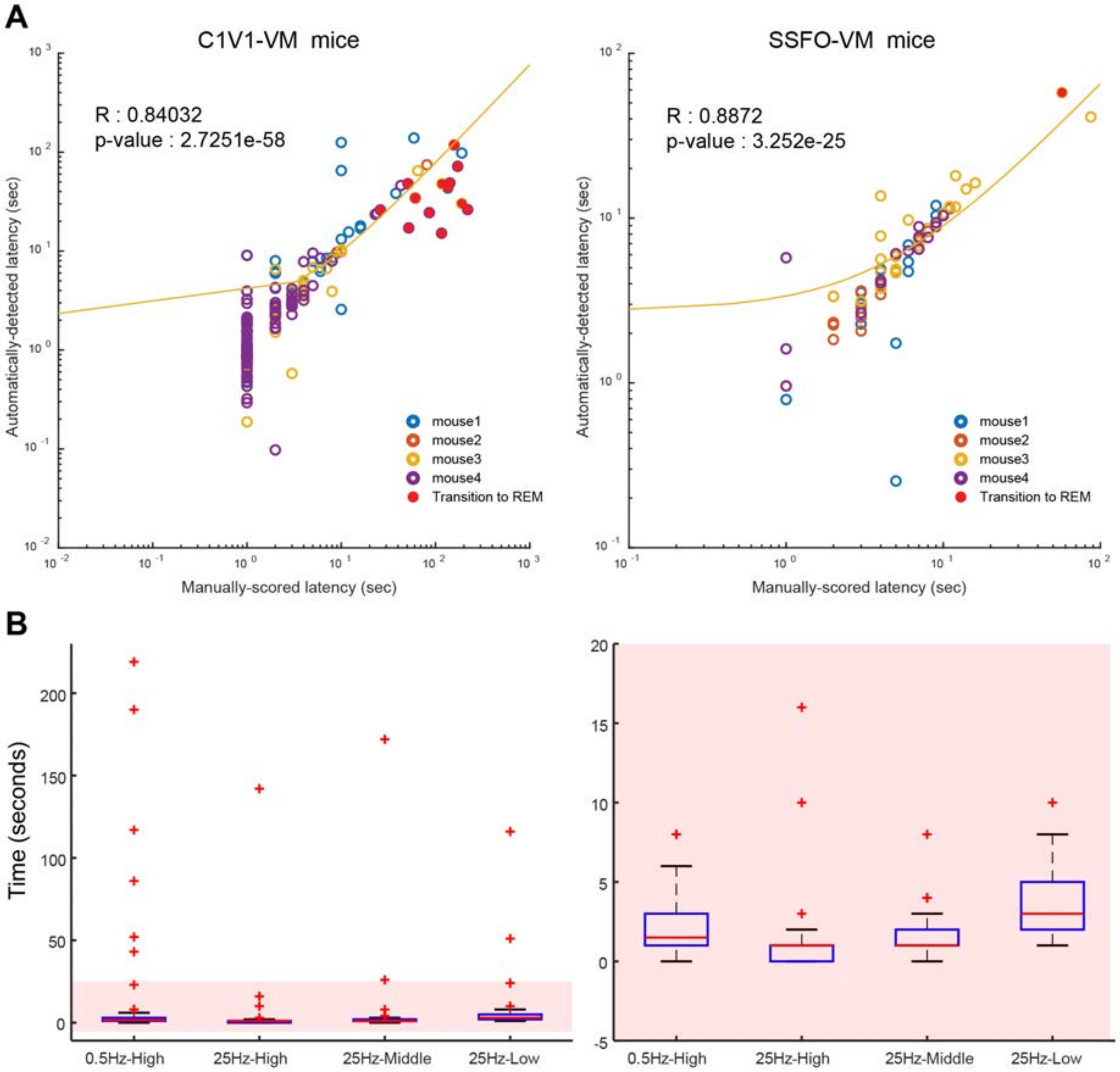

Supplementary Figure 8. Validation of manual scoring and effects of different intensity of laser stimulation. (A) Correlation between latency to arousal defined automatically (x axis) and manually using visual scoring (y axis). In the automatic detection, awakening was defined as the moment when the whisker EMG signal exceeds by more than 10 std the mean value calculated during 10 secs of baseline NREM sleep. The correlation is shown for optogenetically-induced arousals in Calb1-Cre C1V1 VM mice (left) and in Calb1-Cre SSFO VM mice (right). (B) Box plots showing the distribution of manually scored latency to arousal from NREM sleep using four different patterns of laser stimulation (0.5 or 25 Hz, at low, medium or high intensity; central mark; median, top and bottom of the box; 25th and 75th percentiles, whiskers extend from minimum to maximum except for outliers, +; outliers). All types of stimulation successfully awakened mice from NREM sleep, with no statistical differences (Wilcoxon rank sum test, p = from 0.33 to 0.67).

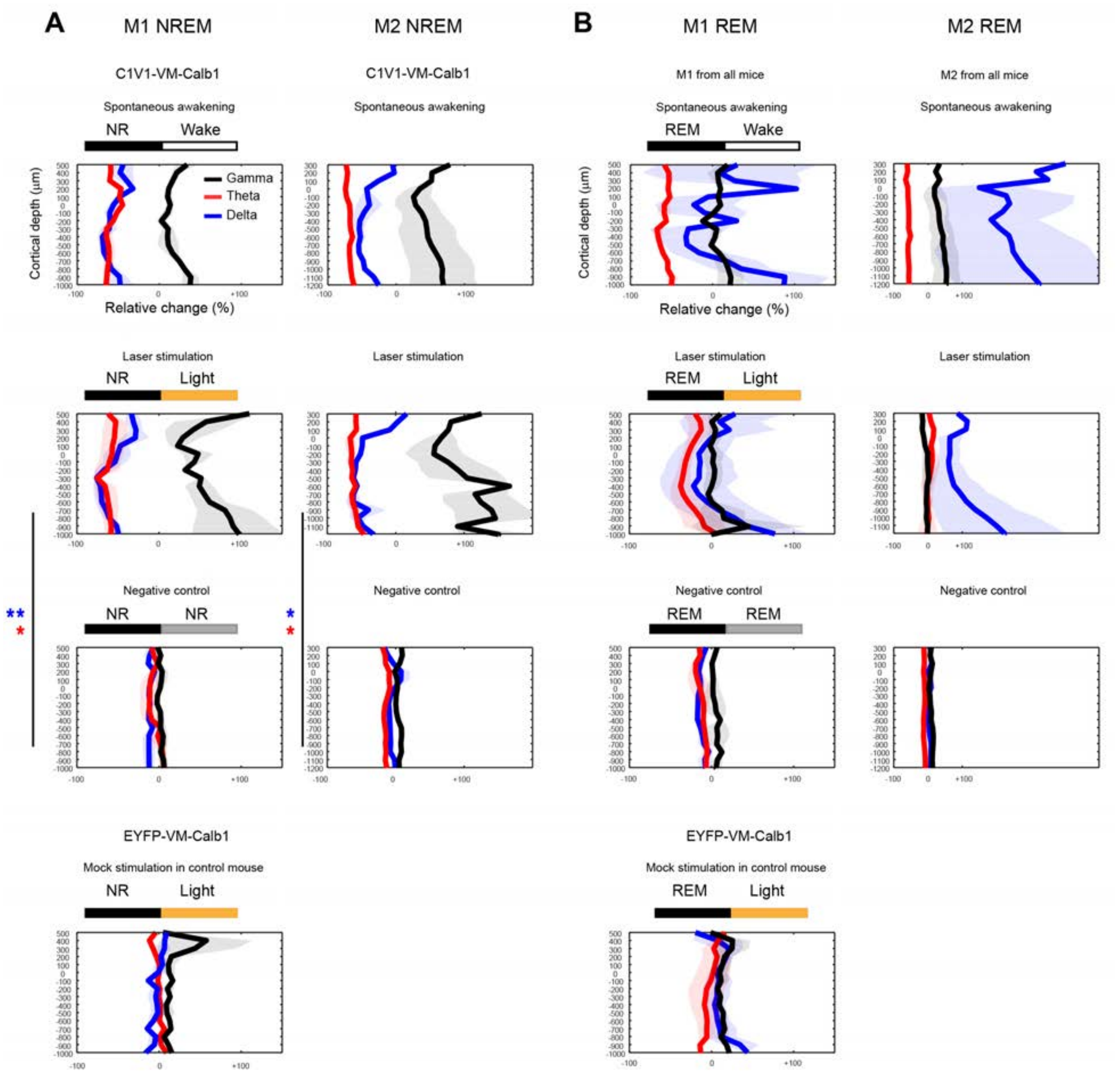

Supplementary Figure 9. Changes in cortical LFP power during spontaneous awakening and during arousals after VM optogenetic stimulation. LFP power changes in M1 and M2 cortex during arousal from NREM sleep (A) and REM sleep (B). Lack of changes after control stimulations are also shown. The Y axis of each panel shows relative cortical depth, with 0 indicating the putative layer III/V boundary. The X axis shows percent change relative to the baseline for SWA (blue, 0.5-4 Hz), theta (red, 6-10 Hz) and gamma activity (black 80-100 Hz). Shaded areas represent std. Asterisks show p-values for the comparisons between laser stimulation and negative control stimulation in the SWA (blue) and theta (red) range. * $p<0.05$, ** $p<0.01$, t-test using the mean of LFP power changes in a deep layer channel (10 secs post-light onset / 10 secs pre-light onset, n=3 for M1 and n=2 for M2 from 4 mice, since one mouse had two cortical probes).

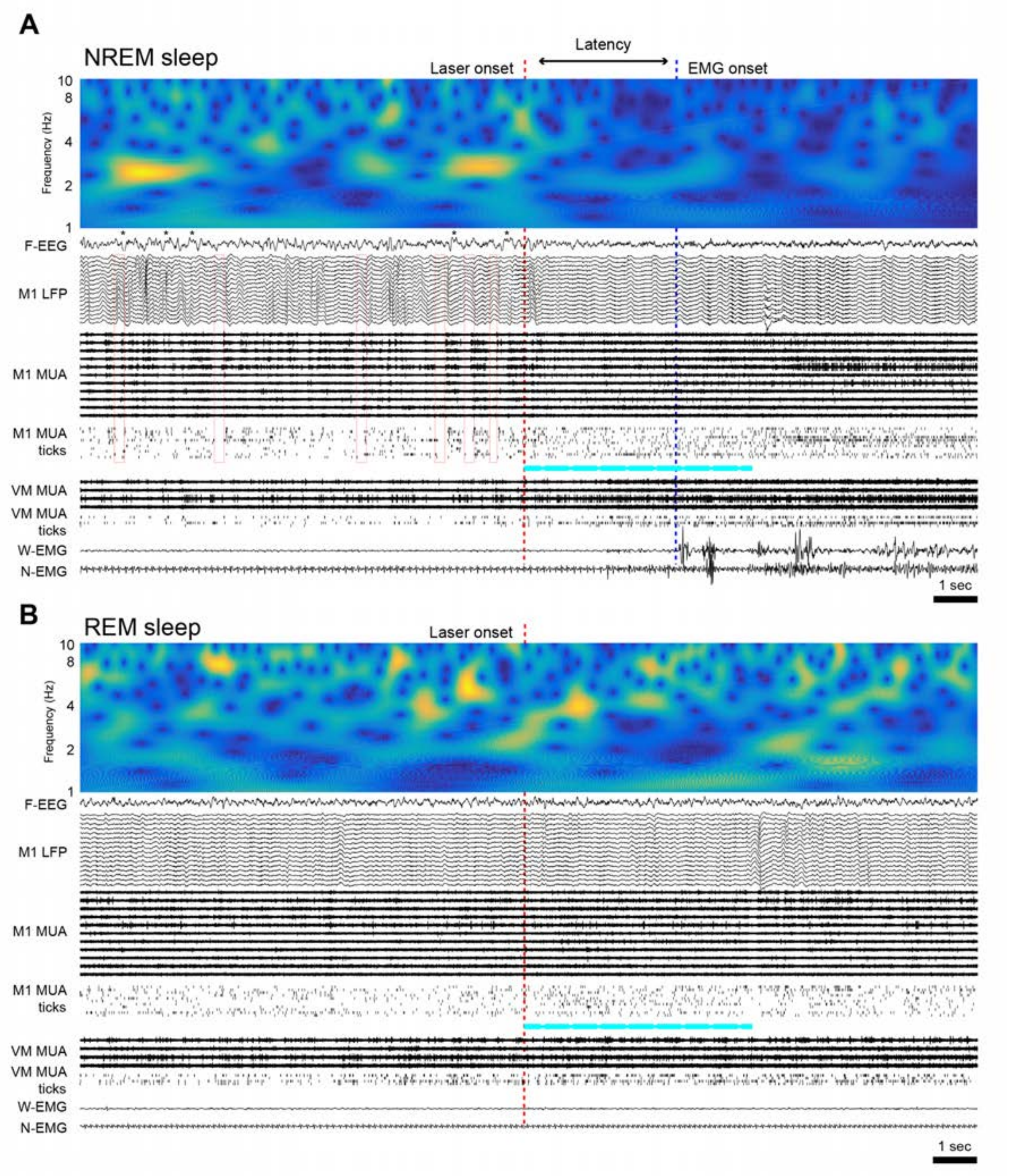

Supplementary Figure 10. SSFO-mediated optogenetic stimulation of VM neurons awakens mice from NREM sleep but not from REM sleep. (A) Example of awakening after laser stimulation of VM in NREM sleep, showing the 10 secs before and after laser onset. Top; EEG power in the SWA (0.5-4 Hz) and theta (6-10 Hz) range decreases a few secs after laser onset. Red open squares identify OFF periods. The blue bar indicates a single laser pulse (5 sec), which strongly drives VM unit firing (VM MUA and ticks, thresholded spikes). The blue dotted line indicates the time of automatically-detected awakening based on whisker EMG signal. (B) Example of VM stimulation in REM sleep.

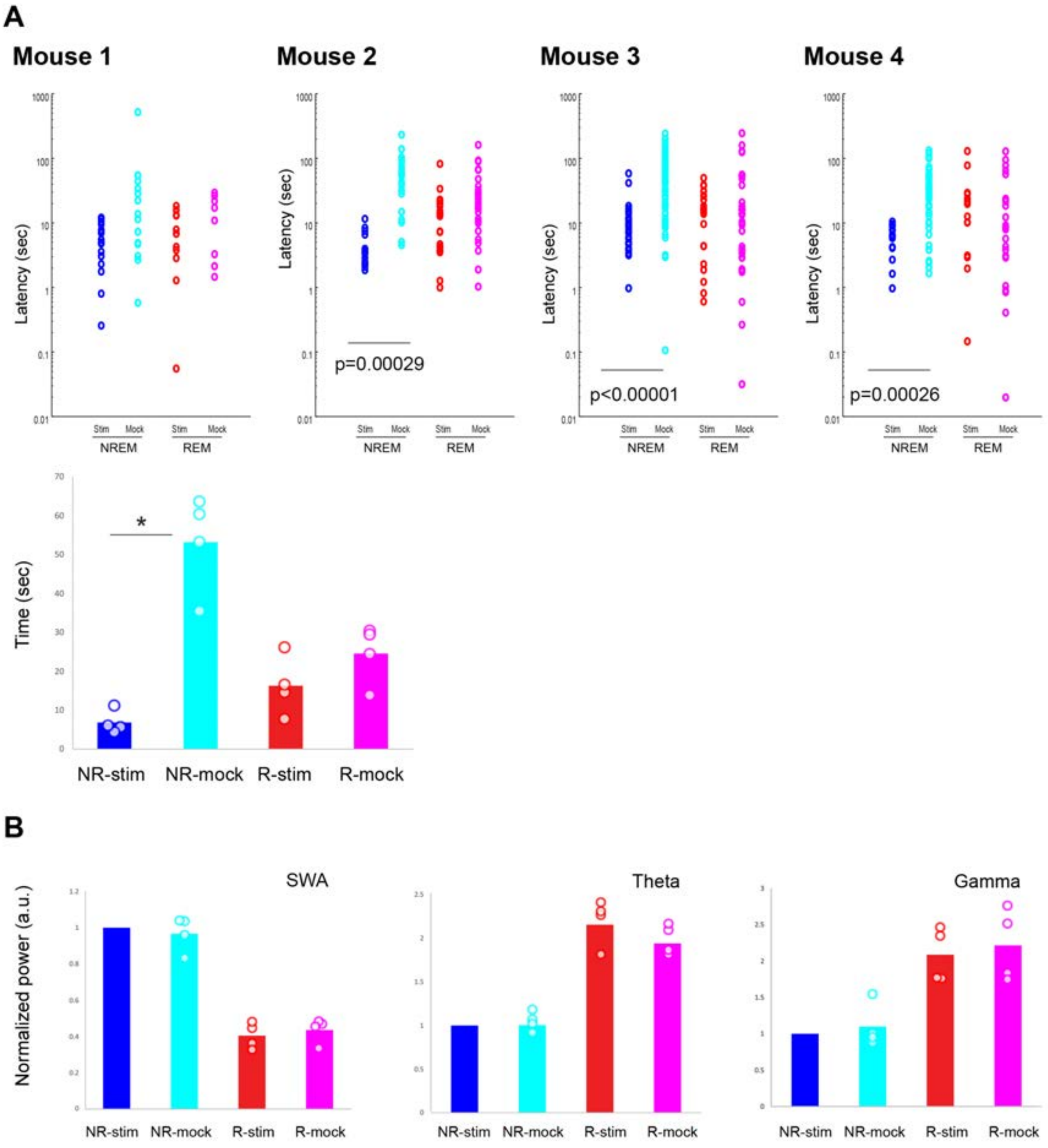

Supplementary Figure 11. Characterization of individual responses to light stimulation in SSFO mice. (A, top) Scatter plots of automatically-detected latency to awakening from light onset of 4 SSFO-VM mice. p values by t-test using all latency values from stimulation and mock are shown for NREM sleep. (A, bottom) Bar plots for the group level analysis: each dot shows the mean latency of one mouse and bars represent mean of the 4 mice. *; $p < 0.005$, paired t-test. (B) EEG power in specific frequency bands during the last 10 secs preceding the stimulation, to show that laser pulses and mock stimulations occurred under consistent brain states. Parietal EEG power was normalized to that of NREM-stim in each frequency band (SWA; 0.5-4 Hz, theta; 6-10 Hz, gamma; 30-100 Hz). Paired t-tests (NR-stim vs. NR-mock; R-stim vs. R-mock), all p-values ≥ 0.1298.

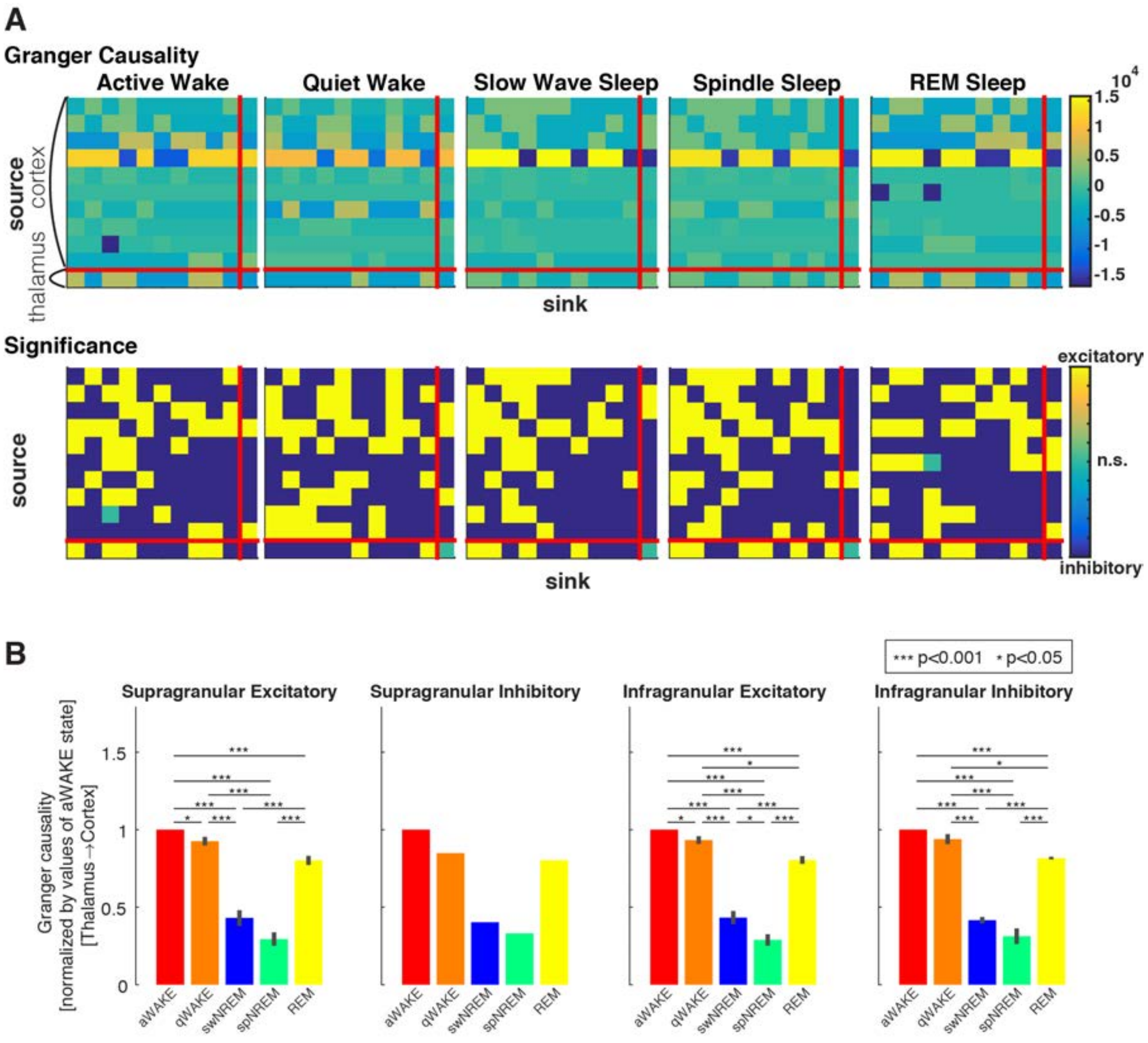

Supplementary Figure 12. Thalamocortical Granger Causality (GC) estimated with spike trains. (A, top) Example of GC matrix for one mouse. Values in each row of the matrix indicate strengths of GC influences from the corresponding source neuron to sink neurons, whereas those in each column shows strengths of GC influences from source neurons to the sink neuron. Positive values indicate excitatory causal influences, while negative values indicate inhibitory influences. The orders of cells in rows and columns are the same, and were determined according to the depths of the recording channels. (A, bottom) A matrix showing statistical significance of GC values. Yellow entries indicate statistical significance of excitatory causal influences, whereas blue entries indicate statistical significance of inhibitory causal influences. Green entries indicate no significant causal influences. (B) The group-mean of GC for all neuron categories. For each thalamocortical neuron pair, absolute values of GC strengths in all conditions were normalized by the value in the active wake state. This normalization was conducted to avoid contamination of the effect of inter-mouse variability in GC strengths on statistical tests. The normalized GC strengths in the same neuron category were averaged to obtain a mean value for that category in each mouse. The group-mean of the normalized GC strengths was compared between all pairs of states for each category by t-test. All error bars show standard errors of GC strengths (n= 7 mice).

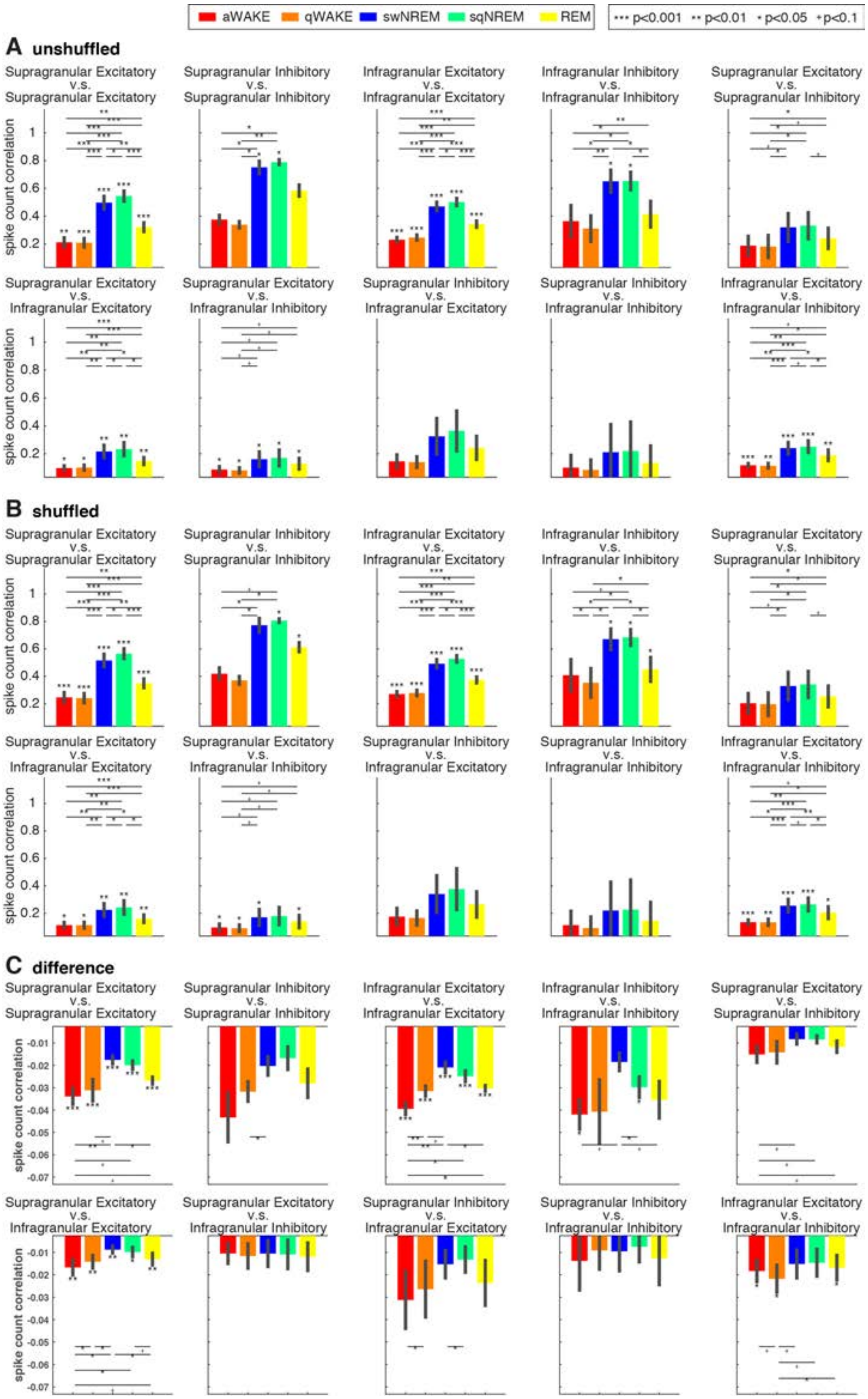

Supplementary Figure 13. Spike count correlation (SCC) analysis with spontaneous spikes. (A) Group-mean values of SCC in five behavioral states, and (B) the corresponding values obtained by permuting inter-spike intervals (ISI) of referential thalamic spikes. (C) The effect of thalamic inputs on SCC calculated by subtracting the group-mean values obtained by permuted ISIs from those obtained by intact ISIs. All error bars show standard errors (n= 7 mice).

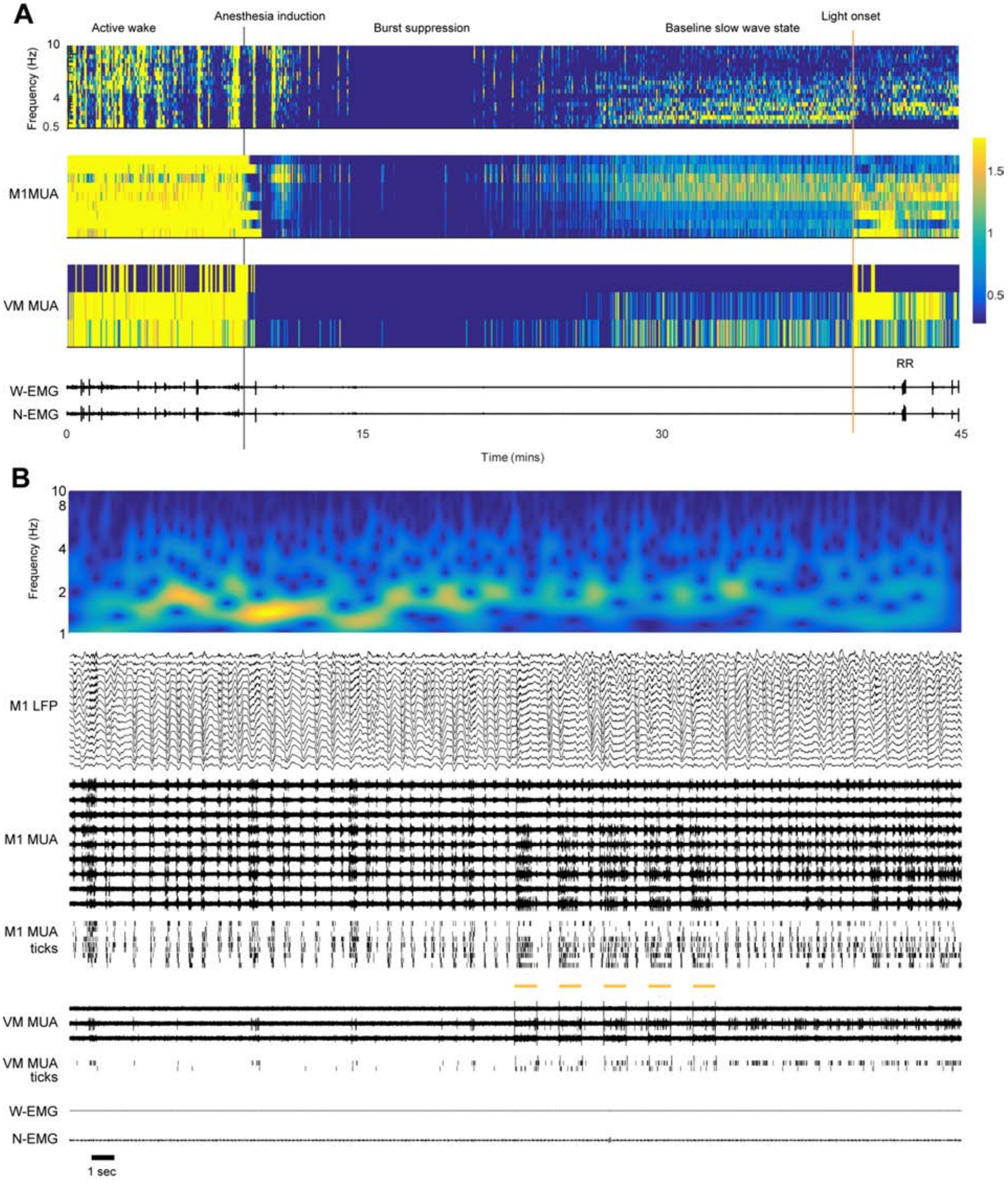

Supplementary Figure 14. Example of C1V1-mediated VM activation under anesthesia. (A) From top: power spectrum in the SWA and theta range (0.5-10Hz), M1 MUA, VM MUA, and EMG signals from whiskers and neck muscles. All signals are normalized to the mean of the plotted 45 mins. (B) As in A (and M1 LFPs) for 40 secs around the time of laser stimulation. The yellow bars indicate when the light is on.

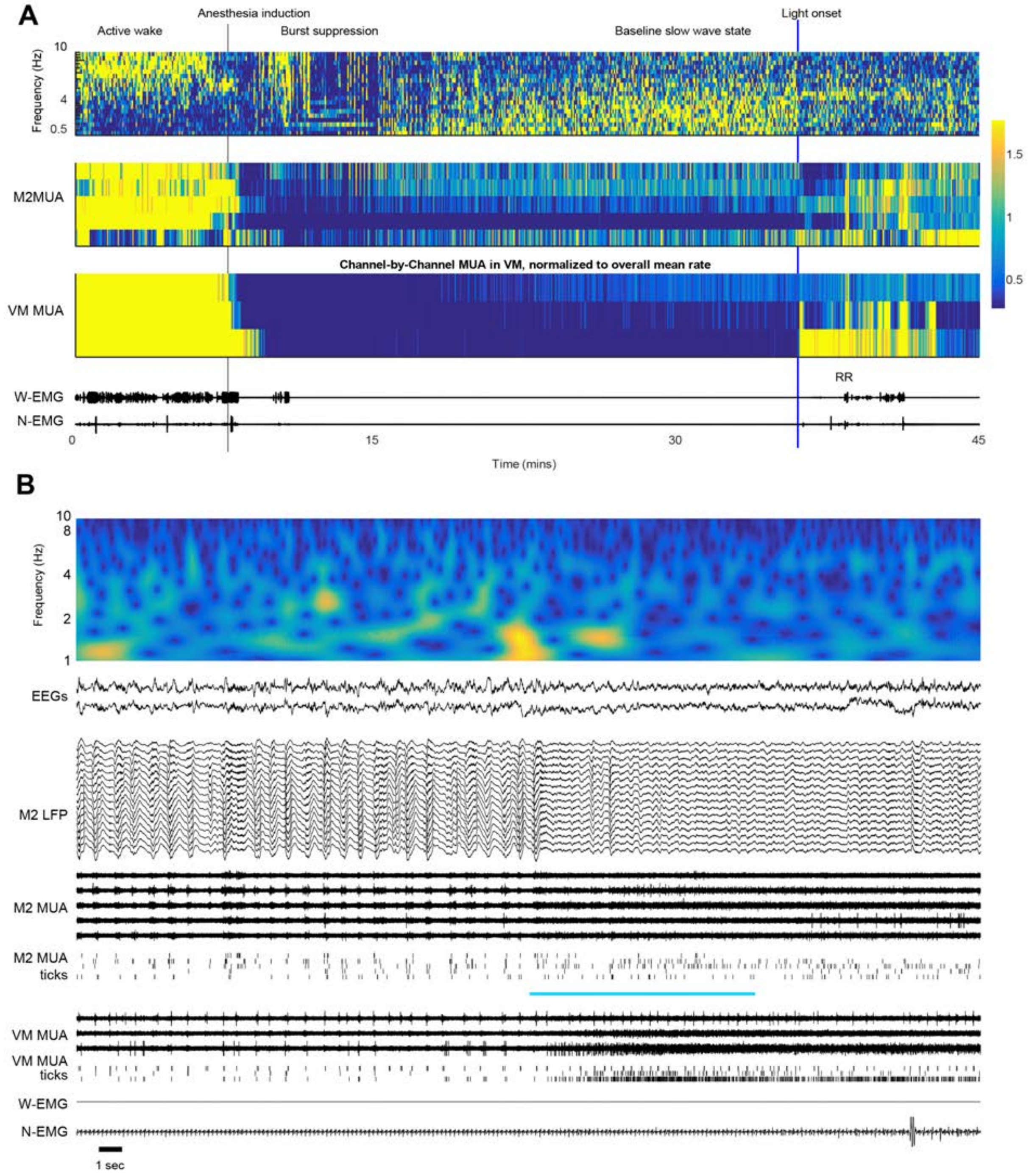

Supplementary Figure 15. Example of SSFO-mediated VM activation under anesthesia. (A) From top: power spectrum in the SWA and theta range (0.5-10Hz), M2 MUA, VM MUA, and EMG signals from whiskers and neck muscles. All signals are normalized to the mean of the plotted 45 mins. (B) As in A (and M2 LFPs) for 40 secs around the time of laser stimulation. The blue bar indicates when the light is on.

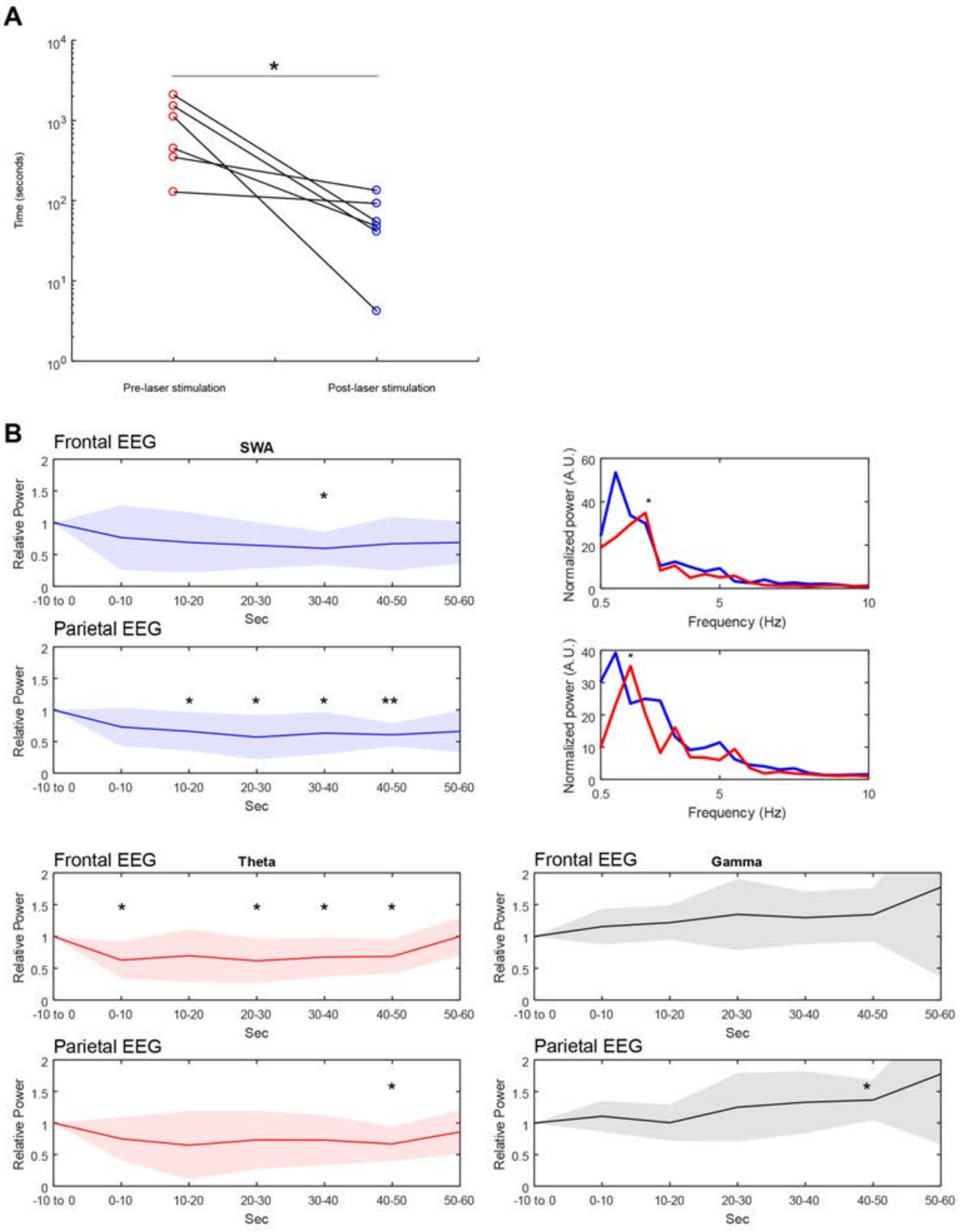

Supplementary Figure 16. Summary of the effects of VM optogenetic stimulation under anesthesia. (A) Automatic analysis of motor activity using the whisker EMG. The Y axis shows the time when the whisker EMG signal exceeded mean + 10 sd of the baseline values calculated during the last 10 secs before laser stimulation. Supra-threshold EMG values were reached significantly faster after laser stimulation as compared to before laser stimulation N=6 mice, * $p < 0.05$, paired t-test. (B) Top panels. SWA power in frontal and parietal EEG decreases after VM stimulation, and the SWA peak shifts to faster frequencies. In the right panel, blue and red lines refer to the 10 secs before and after light onset, respectively. Bottom panels. VM stimulation also decreases the EEG theta power and tends to increase the EEG gamma power. Shaded areas represent std. N=6, * $p < 0.05$, ** $p < 0.01$, paired t-test.

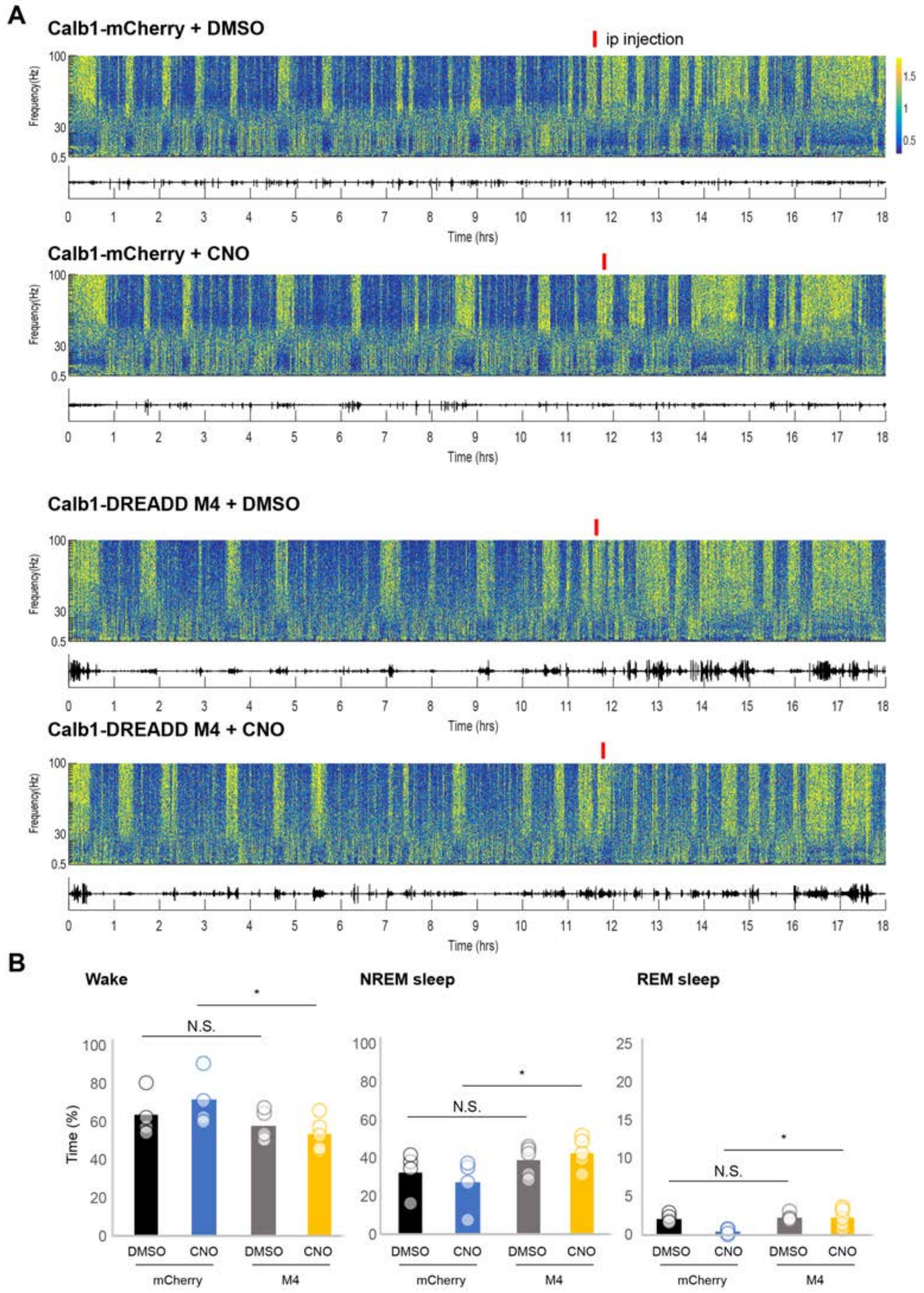

Supplementary Figure 17. DREADD-mediated VM inactivation during the dark phase. (A) Relative EEG power in frontal cortex, normalized to the 18 h mean for each frequency (Y axis, 0.5-100 Hz, 1 Hz bin) and neck EMG are shown. Color bar on the right represents relative changes for all four panels. Experimental conditions are indicated above each panel and red bars show i.p. injection timing. (B) Quantification of each vigilance state. Each dot shows the percentage of the vigilance state during the first 6 hours after i.p injection in each experiment. Bars show mean of 5 experiments in 5 mice. *; wake p = 0.0074; NREM sleep, p = 0.0133; REM sleep, p = 0.0001, see Methods LME model for details.

Supplementary Table 1

Table 1 proportion of thalamocortical neuron pairs showing significant peaks in the correlogram

Mouse	layer	cell	active wake (%)	active wake (%, excl. pairs showing peaks preceding thalamic spikes)	quiet wake (%)	quiet wake (%, excl. pairs showing peaks preceding thalamic spikes)	slow wave NREM sleep (%)	slow wave NREM sleep (%, excl. pairs showing peaks preceding thalamic spikes)	spindle NREM sleep (%)	spindle NREM sleep (%, excl. pairs showing peaks preceding thalamic spikes)	REM sleep (%)	REM sleep (%, excl. pairs showing peaks preceding thalamic spikes)	# cortical neurons	# thalamic neurons	# all neuron pairs
1	supragranular	Excitatory	0	0	14.3	14.3	28.6	14.3	0	0	0	0	7	1	7
		Inhibitory	0	0	0	0	0	0	0	0	0	0	1	1	1
	infragranular	Excitatory	0	0	0	0	6.7	0	6.7	6.7	0	0	15	1	15
		Inhibitory	0	0	0	0	100	0	100	0	0	0	1	1	1
2	supragranular	Excitatory	0	0	0	0	0	0	0	0	0	0	4	1	4
		Inhibitory	NaN	NaN	NaN	NaN	NaN	NaN	NaN	NaN	NaN	NaN	0	1	0
	infragranular	Excitatory	0	0	0	0	0	0	16.7	16.7	0	0	6	1	6
		Inhibitory	NaN	NaN	NaN	NaN	NaN	NaN	NaN	NaN	NaN	NaN	0	1	0
3	supragranular	Excitatory	0	0	0	0	0	0	0	0	0	0	7	8	56
		Inhibitory	NaN	NaN	NaN	NaN	NaN	NaN	NaN	NaN	NaN	NaN	0	8	0
	infragranular	Excitatory	2.5	2.5	0	0	0	0	5	5	0	0	5	8	40
		Inhibitory	0	0	0	0	12.5	12.5	0	0	0	0	1	8	8
4	supragranular	Excitatory	0	0	8.3	8.3	0	0	8.3	0	0	0	6	2	12
		Inhibitory	NaN	NaN	NaN	NaN	NaN	NaN	NaN	NaN	NaN	NaN	0	2	0
	infragranular	Excitatory	0	0	0	0	0	0	0	0	0	0	8	2	16
		Inhibitory	NaN	NaN	NaN	NaN	NaN	NaN	NaN	NaN	NaN	NaN	0	2	0
5	supragranular	Excitatory	0	0	0	0	0	0	0	0	0	0	2	2	4
		Inhibitory	NaN	NaN	NaN	NaN	NaN	NaN	NaN	NaN	NaN	NaN	0	2	0
	infragranular	Excitatory	10	10	0	0	10	10	10	10	10	10	5	2	10
		Inhibitory	NaN	NaN	NaN	NaN	NaN	NaN	NaN	NaN	NaN	NaN	0	2	0
6	supragranular	Excitatory	0.7	0.7	0	0	16.2	2.9	1.5	1.5	0	0	8	17	136
		Inhibitory	0	0	0	0	61.8	8.8	50	0	2.9	0	2	17	34
	infragranular	Excitatory	0	0	0	0	0	0	5.9	3.9	0	0	3	17	51
		Inhibitory	0	0	0	0	26.5	0	11.8	0	2.9	2.9	2	17	34
